# A novel sulfur‐containing photoinitiator based on benzophenone derivatives for rapid photopolymerization

**DOI:** 10.1002/smo.20240065

**Published:** 2025-03-28

**Authors:** Yu Li, Wenzheng Li, Saihe Yang, Lu Li, Shikun Song, Yuting Tang, Han Han, Zhenyue Duan, Yangyang Xin, Jiangli Fan, Pengzhong Chen, Xiaojun Peng

**Affiliations:** ^1^ State Key Laboratory of Fine Chemicals Dalian University of Technology Dalian China; ^2^ Key Lab of Organic Optoelectronics and Molecular Engineering of Ministry of Education Department of Chemistry Tsinghua University Beijing China; ^3^ Ningbo Institute of Dalian University of Technology Ningbo China

**Keywords:** benzophenone, hydrogen abstraction, lysis, photoinitiators, photopolymerization

## Abstract

In this study, a series of benzophenone (BP)‐based photoinitiators (PIs), designated as BP‐Sn, were synthesized by the integration of small molecule hydrogen donors into the BP scaffold. This design aims to minimize reliance on co‐initiators, reduce potential odors and toxicities, and enhance initiation efficiency of PIs under LED irradiation. Additionally, the incorporation of thioether linkages imparts a distinctive dual functionality to PIs, enabling them to exhibit both hydrogen‐abstracting (Type II) and bond‐breaking (Type I) characteristics. The photophysical and photoinitiation properties of BP‐Sn were evaluated using electron paramagnetic resonance, UV‐visible spectrophotometry, and real‐time Fourier‐transform Infrared spectroscopy. The findings demonstrate that BP‐Sn exhibit remarkable efficacy as a single‐component PI for free‐radical polymerization, achieving a final conversion efficiency approaching 95% and a photoinitiation rate exceeding 20% s^−1^ for the polymerization of tripropylene glycol diacrylate without the need for additional co‐initiators. Furthermore, the polymerization experiment revealed that exposure to 365 nm LED light for just 2 min results in the formation of a colorless and well‐defined patterned texture, with a thickness of 5 mm and a diameter of 7 cm, demonstrating its potential practical applications.

## Graphical abstract

This study develops a series of unimolecular photoinitiators, BP‐S1‐5, by incorporating hydrogen‐donating groups into a benzophenone framework. This method reduces the reliance on co‐initiators, and enables BP‐S1‐5 with efficient photoinitiation and excellent molding capability.

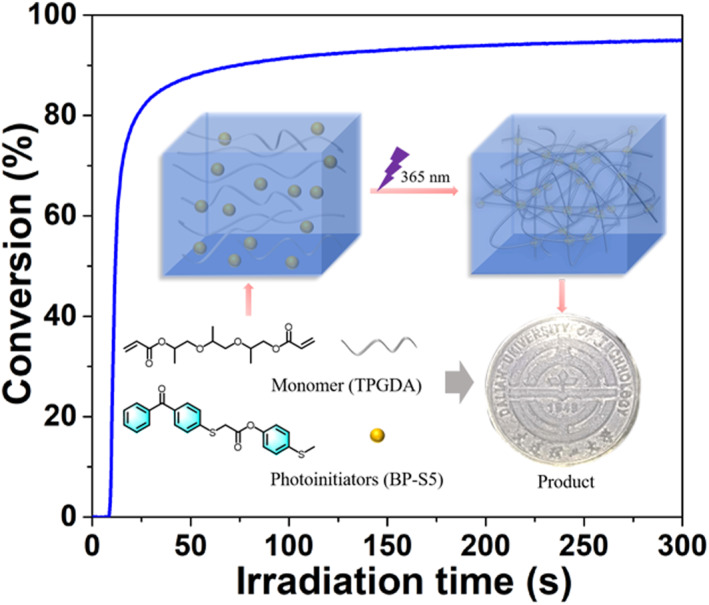

## 1 INTRODUCTION

Polymer materials are widely utilized across various sectors, including semiconductors, agriculture, aerospace, and medicine, owing to their mechanical strength, thermal stability, electrical insulation properties, and ease of processing.[^1^, ^2^, ^3^, ^4^, ^5^] Photopolymerization has become an efficient and eco‐friendly alternative to traditional thermal‐based polymer synthesis methods,^[6]^ facilitating the conversion of monomers or oligomers into solid products via exposure to light or electron beams irradiation.^[7]^ Photoinitiator (PI) plays a crucial role in this process, as they generate active species such as free radicals upon light exposure. Currently, the transition from ultraviolet mercury lamp to energy‐efficient light‐emitting‐diodes (LEDs) emitting visible light has created a demand for the development of innovative PIs that are specifically optimized for irradiation with visible LED light.[^8^, ^9^]

PIs are classified into two categories: Type I, which generates free radicals through lysis, and Type II, which generates free radicals via hydrogen abstraction.^[10]^ Specifically, Type I PIs are characterized by their capacity to produce free radicals through the cleavage of weak chemical bonds, offering high polymerization efficiency; but they are often unstable when exposed to light.^[11]^ In contrast, Type II initiators, which incorporate hydrogen acceptors and co‐initiators acting as hydrogen donor, generate free radicals through electron or proton transfer mechanisms. This process ensures the precise and selective production, and Type II PIs generally exhibit better stability against light and environmental factors compared to their Type I counterparts.[^12^, ^13^, ^14^, ^15^]

To date, various systems based on benzophenone (BP),[^16^, ^17^, ^18^] chalcone,[^19^, ^20^] thioxanthone,[^21^, ^22^] BODIPY,^[23]^ coumarin,^[24]^ naphthalimide^[25]^, and oxime esters^[26]^ have been developed. Among these, BP‐based PIs are particularly notable for their widespread use as hydrogen‐abstraction initiators, attributed to their strong UV absorption, straightforward synthesis, and cost‐effectiveness.^[27]^ At present, the limited reactivity of BP necessitates the inclusion of co‐initiators in BP‐based systems to improve the efficacy of polymerization reactions. However, the initiation efficiency of these multi‐component systems is often compromised by the cage effect and the viscosity of the system.^[28]^ Furthermore, significant yellowing and toxicity migration may occur as a result of the migration of small molecules such as PIs or co‐initiators, during polymerization process.[^29^, ^30^, ^31^] For example, the frozen noodle contamination incident that occurred in Belgium in 2011 was initiated by the transfer of BP from the printing ink used on the noodle packaging into the noodles It notably suggests the necessity for ongoing intensive research endeavors aimed at enhancing the performance of PIs.

In this study, we developed a series of unimolecular PIs, designated BP‐S1‐5, by incorporating small‐molecule hydrogen‐donating groups (alkoxy, aminoalkyl, and alkylthio) onto a BP framework. This design strategy aims to minimize the reliance on co‐initiators, thereby mitigating issues such as yellowing migration, while investigating the impact of varying structures of hydrogen‐donating groups on photoinitiation efficacy. Notably, the introduction of a thioether bond, characterized by a lower C‐S bond energy, is expected to undergo cleavage upon photoexcitation. This innovative molecular design seeks to combine the stability and selectivity of Type II PIs with the high reactivity typically observed in Type I PIs. The BP‐S1‐5 compounds were characterized using UV‐Vis spectroscopy, electrochemical analysis, thermogravimetric analysis (TGA), and Electron paramagnetic resonance (EPR). Their photoreactivity was examined by exposing polymerization of tripropylene glycol diacrylate (TPGDA) to LED@365‐nm, and their photoinitiation capabilities were evaluated using Fourier‐transform infrared (RT‐FTIR) spectroscopy. The findings showed a final conversion efficiency approaching 95% and a photoinitiation rate exceeding 20% s^−1^ for the polymerization of TPGDA without additional co‐initiators. In comparison, the commercial BP under the identical experimental conditions demonstrated only 85% conversion efficiency and a maximum photoinitiation rate of 9% s^−1^.

## 2 EXPERIMENTAL

### 2.1 General information and materials

4‐Chlorobenzophenone, mercaptoacetic acid, *N, N*‐dimethylacetamide (DMAC), sodium hydroxide, *N, N′*‐dicyclohexylcarbodiimide (DCC), 4‐dimethylaminopyridine (DMAP), 4‐methoxyphenol, 3,4‐dimethoxyphenol, 3,4‐dimethoxybenzeneethanol, 4‐methylthiophenol, 4‐dimethylaminophenol, *N*‐phenylglycine (NPG), and *α*‐phenyl‐*N*‐tert‐butylnitrone (PBN) are purchased from Energy Chemical. Hydroxyethyl acrylate (HEA), tripropylene glycol diacrylate (TPGDA), 1,6‐hexanediol diacrylate (HDDA), and trimethylolpropane triacrylate (TMPTA) were purchased from Aladdin Reagent Co. Ltd, and were purified with a neutral alumina column to eliminate inhibitors prior to their applications. Other chemicals were used as received, without further purification, unless specified otherwise.

Nuclear magnetic resonance (NMR) spectra were acquired utilizing the Bruker Avance II 400 and Bruker AVANCE NEO 600M spectrometers. Mass spectrometric data were collected using the G6230B instrument employing electrospray ionization (ESI‐MS). The absorption and emission spectra for all compounds were recorded using a Lambda 35 UV‐visible spectrophotometer (PerkinElmer) and a NanoDrop 3300 fluorescence spectrophotometer, respectively. The fluorescence quantum yield was determined using a HAMAMATSU absolute fluorescence quantum yield spectrometer. The polymerization kinetic curves were measured through real‐time FT‐IR spectroscopy (FT‐IR) using a Nicolet 5700 instrument. Radical species were characterized via EPR using Bruker A200–9.5/12 instrument. The thermal stability of the synthesized PIs was analyzed through TGA with a TGA/SDTA851e instrument.

### 2.2 Spectra characterization

The five PIs in anhydrous acetonitrile solutions were formulated with concentration gradients ranging from 1 to 5 × 10^−5^ mol L^−1^. The absorbance of these solutions was measured using a UV‐Vis spectrophotometer. In accordance with the Lambert–Beer law, as delineated in Equation (1), the molar extinction coefficients of the photoinitiator molecules at designated wavelengths were calculated.

(1) ε=A/cL

Where *A* signifies the absorbance of the solution; *ε* (M^−1^ cm^−1^) denotes the molar extinction coefficient; *c* (mol L^−1^) indicates the concentration of the solution; and *L* (cm) refers to the thickness of the cuvette which is set to 1 cm in this experiment.

The fluorescence emission spectra of the prepared PIs solutions at a concentration of 5 × 10^−5^ M, were examined using an excitation wavelength of 365 nm and slit width of 5 nm within the optical path.

### 2.3 Photolysis studies

Photolysis studies were conducted on BP‐Sn acetonitrile solutions with concentrations of 1 × 10^−5^ mol L^−1^ using the LED@365‐nm (100 mW⋅cm^−2^) as the irradiation source under ambient conditions. Variations in absorbance were recorded at specified time intervals.

### 2.4 Cyclic voltammetry analysis

The BP‐S1‐S5 in acetonitrile solutions were prepared at a concentration of 1 × 10^−3^ mol L^−1^. Subsequently, tetrabutylammonium hexafluorophosphate was added into the solution as an electrolyte at a concentration of 0.1 mol L^−1^. Prior to conducting measurement, the solution was purged with nitrogen for a duration of 15 min to mitigate the influence of oxygen. The oxidation potential (E_ox_) and reduction potential (E_red_) of the PIs were determined using cyclic voltammetry on an electrochemical workstation. A platinum wire electrode was utilized as the working electrode, a glassy carbon electrode served as the counter electrode, and a silver/silver chloride (Ag/AgCl) electrode functioned as the reference electrode. The scan rate was 0.05 V s^−1^. The Gibbs free energy change for electron transfer of these PIs, denoted as ΔG_et_, was calculated using the Rehm‐Weller equation, as represented in Equation (2).

(2) $$∆G_{et}=E_{ox}-E_{red}-E^{*}+C$$

Where *E*_ox_ represents the oxidation potential of the electron donor; *E*_red_ denotes the reduction potential of the electron acceptor; the parameter E* represents the energy of the singlet excited state of the photoinitiator, which is calculated by determining the intersection point of the normalized absorption and emission spectra; *C* denotes the electrostatic interaction energy of ion pair that is initially formed, which is typically considered negligible in polar solvent environments.

### 2.5 Thermal stability testing

A specific amount of the photoinitiator molecules was accurately measured and analyzed using a thermogravimetric analyzer. The temperature program was set to increase from 50 to 600°C at a heating rate of 10°C ⋅per minute under an argon atmosphere.

### 2.6 Photopolymerization procedures

Photocurable formulations were prepared by thoroughly mixing the requisite components through stirring until homogenous resin mixtures were achieved. These photocurable formulations were sandwiched between two potassium bromide (KBr) tablets (thickness = 2 mm) to facilitate the free radical polymerization (FRP) process. During irradiation, the reduction in the double bond for the acrylate functional group was monitored using the Nicolet 5700 FT‐IR spectrometer. The conversion rates of different groups were calculated using Equation (3).

(3) $$\text{Conversion}\%=\left(1-S_{t}/S_{0}\right)\times 100\%$$

where *S*_*t*_ corresponds to the characteristic absorbance peak area observed at 810 cm^−1^ which corresponds to the double bond at specific time *t*, and *S*_0_ represents the initial area of this peak.

### 2.7 Electron paramagnetic resonance (EPR) experiments

The EPR experiments were performed in tert‐butylbenzene solutions that contain PIs at a concentration of 1 × 10^−3^ mol L^−1^, *N*‐phenylglycine (NPG) as a co‐initiator at a concentration of 3 × 10^−3^ mol L^−1^, and phenyl‐*N*‐tert‐butylnitrone (PBN) as a radical scavenger at a concentration of 5 × 10^−3^ mol L^−1^. In a dark chamber, the solution was bubbling with nitrogen for 15 min to eliminate dissolved oxygen. Subsequently, the solution was transferred into a capillary tube, and subjected to a 365 nm LED lamp for a duration of 3 min to facilitate EPR analysis.

### 2.8 Migration testing

The synthesized photoinitiator BP‐Sn and the monomer TPGDA were combined in a weight ratio of 0.3: 99.7 through a stirring process. Subsequently, the resulting photosensitive liquid was transformed into a cylindrical silicone mold with a diameter of 2 cm and a height of 5 mm, and then covered with a PET film to prevent air exposure. The mixture underwent polymerization under LED@365‐nm irradiation, yielding the polymerized samples. Following this, the samples were rinsed with anhydrous ethanol and dried before being immersed in 50 mL of anhydrous acetonitrile solution. Samples were taken at various soaking durations and placed in cuvettes for absorbance measurement. The quantity of the migrated photoinitiator was calculated using Equation (4).

(4) $$\text{Migration}\;\text{ratio}=\left(A\times M\times V_{\text{solution}}\right)/\;\left(\varepsilon \times b\times m\times \omega \right)$$

Where *A* represents the absorbance; *M* is the molar mass of the photoinitiator; *V*_solution_ refers to the total volume of the solution, consistently set at 50 mL across all instances; *ε* represents the molar absorptivity of the photoinitiator in the solution; *b* indicates the optical path length, which remains constant at 1 cm for all scenarios; *m* represents the mass of the cured film, and *ω* denotes the mass fraction of the photoinitiator added into the film, maintained at 0.3 wt% in all cases.

### 2.9 Curing application testing

PIs were mixed with the TPGDA monomer at a weight ratio of 0.1: 99.9 via stirring until a homogeneous mixture was achieved. After degassing to remove any trapped air bubbles, the photosensitive liquid was transferred into specially designed silicone molds. The mixture was then subjected to exposure from LED@365‐nm (100 mW cm^−2^) for a duration of 3 min to commence the curing process.

## 3 RESULTS AND DISCUSSION

### 3.1 Molecular design and synthesis

**FIGURE 1:**
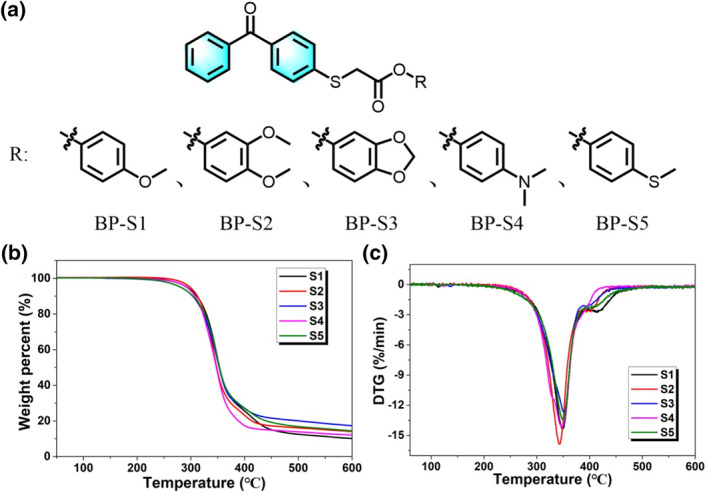
(a) The molecular structures of BP‐Sn, (b) TGA results for BP‐Sn, and (c) differential thermal analysis (DTG) results for BP‐Sn.

Benzophenone was selected as the primary molecular scaffold for the preparation of PIs due to its amenability to structural modification, cost‐effectiveness, and robust hydrogen‐abstracting capabilities. To improve its photoinitiation efficacy, a sulfur ether bond was introduced, characterized by relatively low bond energy and a propensity for cleavage upon photoexcitation. This modification was expected to confer upon the PIs a high reactivity associated with cleavage‐type I, alongside the high stability, and selectivity of hydrogen abstraction‐type II. Additionally, incorporating an ester group was intended to enhance the compatibility of the PIs with acrylate monomers, which was essential for maintaining the stability of formulations and facilitating efficient photopolymerization reactions. Furthermore, various hydrogen‐donating groups, including alkoxy and aminoalkyl, were connected to the BP backbone to form unimolecular Type II PIs (Figure 1a). These groups were selected due to the presence of lone pair electrons and active hydrogen atoms. These characteristics enable the generation of highly reactive free radicals through processes such as electron transfer and proton transfer. The structure‐performance relationship was investigated by assessing the impact of different substituents on the photoinitiator’s initiating capability. The target molecules, designated as BP‐Sn (*n* = 1–5), were synthesized through a two‐step reaction with total yields from 20% to 30% (Supporting Information S1: Scheme S1), and their structures were characterized using ^1^H NMR, ^13^C NMR, and high‐resolution mass spectrometry (Supporting Information S1: Figures S1–S15). BP‐Sn demonstrated exceptional thermal stability, with a weight loss rate of only about 1% when subjected to heating at 250°C, as examined by TGA (Figure 1b,c, Supporting Information S1: Figure S16 and Table S1). Furthermore, the samples were preserved following synthesis without any light protection. The consistency in the ^1^H NMR spectra of the samples of the newly synthesized smaples, as well as those stored for one‐year, confirmed their stability (Supporting Information S1: Figure S16f).

### 3.2 Spectroscopy and photophysics properties of BP‐Sn

**FIGURE 2:**
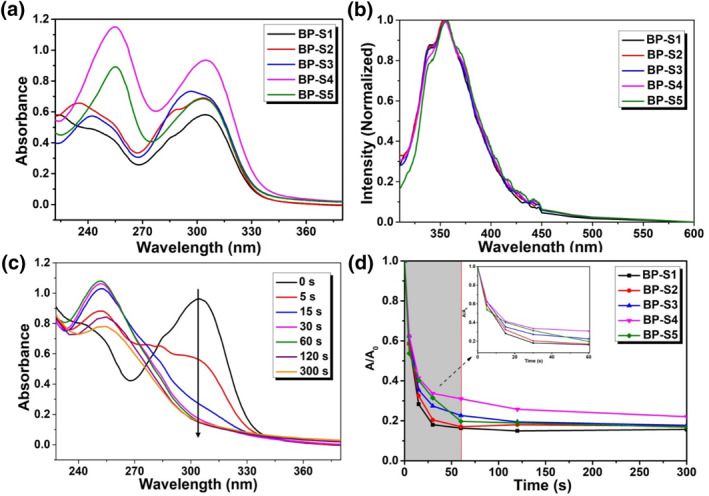
(a) UV‐Vis absorption spectra and (b) fluorescence spectra of BP‐Sn in anhydrous acetonitrile solutions (concentration, 5 × 10^−5^ mol L^−1^; *λ*_ex_ = 300 nm), (c) The steady‐state photodegradation spectra of BP‐S1 in anhydrous acetonitrile over a duration of 300 s, (d) The changes in the absorption spectra of the five PIs as a function of illumination time.

The prepared PIs in anhydrous acetonitrile solutions displayed dual absorption bands within the ranges of approximately 220–270 nm and 280–340 nm, respectively (Figure 2a and Supporting Information S1: Figure S17). The absorption in the lower range was ascribed to the electronic transitions of carboxylic ester moieties, with the absorption maxima being affected by the substituents on benzene ring. These maxima gradually red‐shifted in the following order: ‐N(CH_3_)_2_ (BP‐S4) > ‐SCH_3_ (BP‐S5) > ‐OCH_2_O‐ (BP‐S3) > 2‐OCH_3_ (BP‐S2) > ‐OCH_3_ (BP‐S1), which is consistent with their electron‐donating capabilities (Supporting Information S1: Table S2). The absorption peaks observed around 300 nm were assigned to the *n*–π* transitions of sulfur‐substituted BP units, which were minimally influenced by the electronic properties of the carboxylic ester moieties due to the lack of conjugation between them.

The fluorescence spectra of BP‐Sn in anhydrous acetonitrile solutions showed identical band shapes with emission maxima at 355 nm (Figure 2b). The fluorescence quantum yields were relatively low, recorded at 0.7%, 0.5%, 0.8%, 0.8%, and 0.8% for BP‐S1 to BP‐S5, respectively. Such low quantum yields are consistent with the known behavior of BP derivatives, which typically have a high efficiency of intersystem crossing (ISC) that outcompete the radiative decay process. The resulting populated triplet state may facilitate the photolysis of BP‐Sn, leading to the generation of the active free radicals that can subsequently initiate photopolymerization reactions.[^32^, ^33^]

We further investigated the feasibility of BP‐Sn as PIs under LED@365‐nm irradiation. Generally, in addition to the light absorbance, the photoinitiation capability of PIs is influenced by several factors beyond light absorbance, including bond dissociation energy, solubility, and reactivity of the active species.[^34^, ^35^, ^36^] Despite the unobvious absorbance of these prepared PIs at 365 nm, they showed varying degrees of photodegradation upon irradiation under LED@365‐nm (100 mW cm^−2^) for 300 s. As the irradiation duration increased, the absorbance intensity around 300 nm, attributed to sulfur‐substituted BP units, gradually diminished, indicating that chemical reactions were occurring within the system and that the PIs were being consumed (Figure 2c,d). For BP‐S1, BP‐S2, and BP‐S3, the absorption peaks at 230 nm intensified and redshifted to approximately 250 nm, suggesting the formation of new photolysis products. In contrast, BP‐S4 and BP‐S5 showed no significant changes (Supporting Information S1: Figure S18). Importantly, BP‐S3 exhibited the highest degradation rate of 3.75 × 10^−6^ mol L^−1^ s^−1^ (Supporting Information S1: Table S3), confirming the efficacy of BP‐Sn as a single‐component PI.

### 3.3 Cyclic voltammetry and gibbus free energy

To assess the thermodynamic viability of intramolecular electron transfer reactions in PIs, we conducted a series of electrochemical tests. All five PIs displayed an irreversible reduction peak in the cathodic (negative potential) region. In the anodic (positive potential) region, BP‐S1, BP‐S2, BP‐S3, and BP‐S5 each exhibited irreversible oxidation peaks, while BP‐S4 showed a reversible oxidation peak with a significantly lower oxidation potential relative to the others (Supporting Information S1: Figure S19). This difference may be attributed to the presence of dimethylamino substituent in BP‐S4, as the amino group is particularly prone to oxidation. According to the intersection of normalized absorption and fluorescence spectra, the singlet state (S1) energies of the PIs were determined to be from 3.95 to 4.06 eV (Supporting Information S1: Figure S20). The calculated Gibbs free energies of electron transfer (ΔG_et_) were found to be negative (Table 1), thereby confirming the thermodynamic feasibility of intramolecular electron transfer occurring between the PIs in their singlet excited states.

**TABLE 1:** Electrochemical experimental results of BP‐Sn.

PI	λ(nm)	E_s_(eV)	E_ox_ ^a^ (V vs. SCE)	E_red_ ^a^ (V vs. SCE)	ΔG_et_(eV)
BP‐S1	321	4.06	1.93	−1.67	−0.46
BP‐S2	324	4.02	1.78	−1.68	−0.56
BP‐S3	324	4.02	1.78	−1.69	−0.55
BP‐S4	330	3.95	1.15	−1.70	−1.10
BP‐S5	322	4.05	1.64	−1.66	−0.75

^a^ E_ox_ and E_red_ are obtained from the cyclic voltammetry curve of BP‐Sn.

### 3.4 Photopolymerization

**FIGURE 3:**
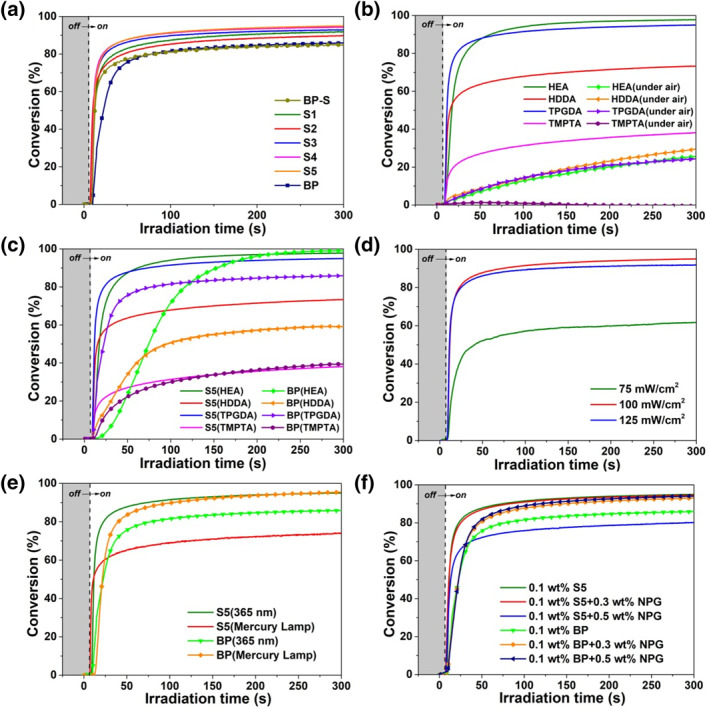
Photopolymerization profiles for various conditions: (a) TPGDA initiated by BP‐Sn and BP (0.1 wt%) under LED@365‐nm (100 mW cm^−2^) irradiation, (b) the polymerization of different monomers initiated by BP‐S5 (0.1 wt%) under LED@365‐nm (100 mW cm^−2^), conducted in both air and an isolated environment, (c) the polymerization of different monomers initiated by BP‐S5 and BP (0.1 wt%) under LED@365‐nm (100 mW cm^−2^), (d) TPGDA polymerization initiated by BP‐S5 (0.1 wt%) under LED@365‐nm with different optical power densities, (e) TPGDA polymerization initiated by BP‐S5 and BP (0.1 wt%) under LED@365‐nm or mercury lamp (100 mW cm^−2^), (f) TPGDA polymerization initiated by BP‐S5 or BP (0.1 wt%) with co‐initiator NPG (0.3/0.5 wt%) under LED@365‐nm (100 mW cm^−2^).

The efficiency of UV curing using BP‐Sn at varying concentrations was firstly investigated. The photopolymerization process was conducted using LED@365‐nm lamp (100 mW⋅cm^−2^). Tripropylene glycol diacrylate (TPGDA) served as the monomer, and the concentrations of PIs were set at 0.1, 0.3, and 0.5 wt%. Notably, at a concentration of 0.1 wt%, the final conversion rate approached 90%. Increasing the concentrations of BP‐Sn to 0.3 and 0.5 wt% resulted in only marginal improvements in the final conversions. Overall, the photopolymerization performance of BP‐Sn was observed to follow the order: BP‐S5 ≈ BP‐S4 > BP‐S2 > BP‐S1 > BP‐S3, which correlated closely with the electron‐donating capabilities of the terminal substituents on the carboxylic esters (Supporting Information S1: Figure S21 and Table S4). Furthermore, at the concentration of 0.1 wt%, the initiation efficiency of BP‐S1‐5 surpassed that of the synthetic intermediate BP‐S and a commercial photoinitiator BP (Figure 3a). This finding underscored the successful development of efficient PIs that operate effectively at low concentrations.

Subsequently, the photopolymerization performances of various monomers including hydroxyethyl acrylate (HEA), tripropylene glycol diacrylate (TPGDA), HDDA, and TMPTA, were evaluated using 0.1 wt% BP‐S5 as PI in an oxygen‐free environment. The photoinitiation rate of HEA was found to be lower than that of TPGDA, HDDA, and TMPTA; however, HEA achieved the highest final conversion of 98% (Figure 3b). The same trend was observed when replacing the photoinitiator with BP, but the reaction rate and maximum polymerization rate were lower than those of BP‐S5 (Figure 3c). This phenomenon was probably ascribed lower number of double bonds in HEA, which could inhibit rapid cross‐linking during polymerization, thereby allowing for a stable and enduring polymerization process. Conversely, TMPTA, which has the highest content of functional groups and viscosity, may restrict the mobility and interactions of active radicals and acrylate double bonds, resulting in the lowest final conversion of only 38.15%. As for TPGDA, it showed faster polymerization rate but yielded a slightly lower final conversion compared to HEA. It is noteworthy that the polymerization also occurred in the presence of air (Figure 3b), suggesting its potential applicability in industrial production, particularly in contexts where precision requirements are not critical.

The photopolymerization efficiencies of 0.1 wt% BP‐S5 in TPGDA were further tested under varying light power densities of 75, 100, and 125 mW⋅cm^−2^ (Figure 3d). An increase in optical power density from 75 to 100 mW⋅cm^−2^ resulted in the enhancement of the final conversion and polymerization rate, which rose from 61%, 7% s^−1^ to 91%, 25% s^−1^, respectively. However, further increasing the power density to 125 mW cm^−2^ produced negligible changes in both the final conversion and polymerization rate. These findings suggest that a power density of 100 mW cm^−2^ may serve as an optimal threshold for photopolymerization within the systems examined, effectively balancing the requisite energy for reaction initiation against the diminishing returns associated with the elevated light intensities.

Additional tests were conducted to assess the photoinitiation efficacy of BP‐S5 under different light sources, with BP serving as a reference (Figure 3e). It revealed that BP‐S5 showed a higher polymerization rate when subjected to mercury lamp irradiation; but the final conversion was only 65%, much smaller than the 95% achieved with LED@365‐nm. The suboptimal performance of BP‐S5 under mercury lamp irradiation is likely attributable to the excessive concentrations of radical produced during the initial irradiation stage which may result in premature termination of the polymerization process, thereby decreasing the final conversion efficiency. In contrast, under LED@365‐nm irradiation, BP‐S5 displayed both a higher polymerization rate and final conversion compared to the commercial BP without a co‐initiator under identical conditions. While BP achieved a comparable final conversion under mercury lamp irradiation, its polymerization rate remained inferior to that BP‐S5.

Given that the majority BP‐based PIs are used in conjunction with co‐initiators, the photoinitiation efficacy under two‐component conditions was tested in the final series of photopolymerization experiments (Figure 3f). The addition of NPG significantly improved the initiating efficiency of BP, with the final conversion increasing from 85% for 0.1 wt% BP alone to about 95% when combined with 0.3/0.5 wt% NPG. In contrast, BP‐S5 showed exceptional initiation efficiency under single‐component conditions, achieving a maximum polymerization rate of 21%⋅s^−1^ and a conversion efficiency of 95%. However, the introduction of 0.5 wt% NPG resulted in a decrease in the final conversion to 80%. This observation suggested that the rapid generation of free radicals in the presence of NPG may lead to significant intermolecular collisions and quenching reactions, thus diminishing the availability of active free radicals necessary for initiating monomer polymerization. Consequently, BP‐S5 does not require the addition of a secondary component to enhance its performance, which is a favorable characteristic for various applications. This is particularly noteworthy as the use of a single‐component system streamlines the formulation and application processes, thereby mitigating complexity and potential challenges associated with the compatibility and mixing of multiple components.

### 3.5 Electron paramagnetic resonance testing and investigation of photoinitiation mechanisms

**FIGURE 4:**
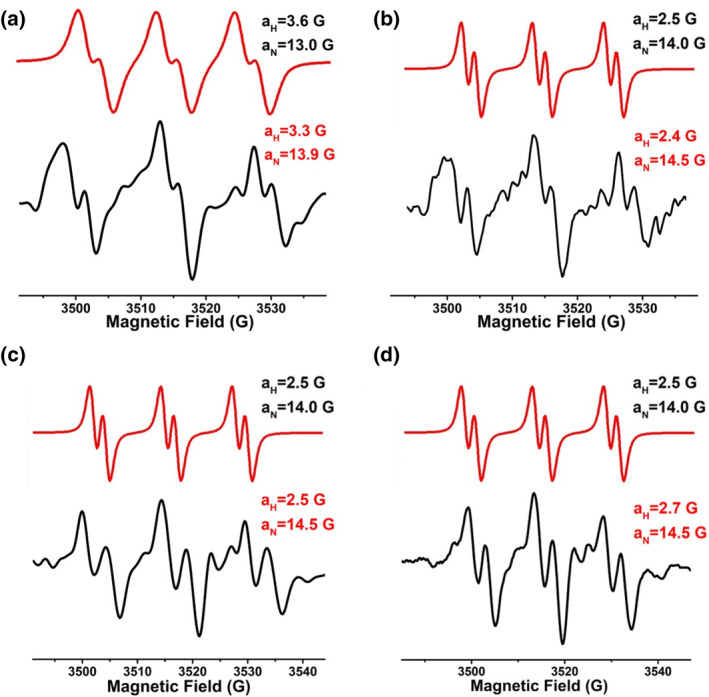
EPR spectra of (a) BP‐S1, (b) BP‐S1/NPG, (c) BP‐S4, (d) BP‐S4/NPG. The red and black lines represent the simulated and experimental data, respectively.

To enhance the understanding of the photoreaction mechanism, EPR measurements were conducted to identify the active species generated during the photoinitiation phase (Supporting Information S1: Figure S22). As shown in Figure 4a,c, the EPR spectra of BP‐S1 and BP‐S4 in tert‐butylbenzene solutions revealed pronounced signals under LED@365‐nm irradiation, suggesting the generation of free radicals in these unimolecular systems. By comparison with the simulated spectra,^[16]^ the signals detected in the BP‐S1 spectrum were assigned to alkoxyl radicals (*a*_H_ = 3.3 G, *a*_N_ = 13.9 G), while those observed in the BP‐S4 spectrum were attributed to aminoalkyl radicals (*a*_H_ = 2.5 G, *a*_N_ = 14.5 G). These radicals correspond to the substituents on the benzene ring, and were generated through intramolecular electron/proton transfer processes within the molecules.

Upon the introduction of the co‐initiator *N*‐phenylglycine (NPG), the EPR spectrum of the BP‐S1/NPG two‐component system exhibited slight variations compared to that of BP‐S1 alone (Figure 4b), whereas the EPR spectrum of BP‐S4/NPG matched well with that of the single‐component system (Figure 4d). In the two‐component BP‐S1/NPG system, in addition to the alkoxyl radicals, it is hypothesized that aminoalkyl radicals were also generated via intermolecular electron/proton transfer between BP‐S1 and NPG. In contrast, BP‐S4/NPG exhibited EPR signals similar to those of BP‐S4 alone, as both systems generated aminoalkyl radicals. The measured EPR spectra of BP‐Sn showed additional peaks relative to the simulated spectra, indicating the formation of other radical species such as sulfur radicals resulting from C–S bond cleavage.

**FIGURE 5:**
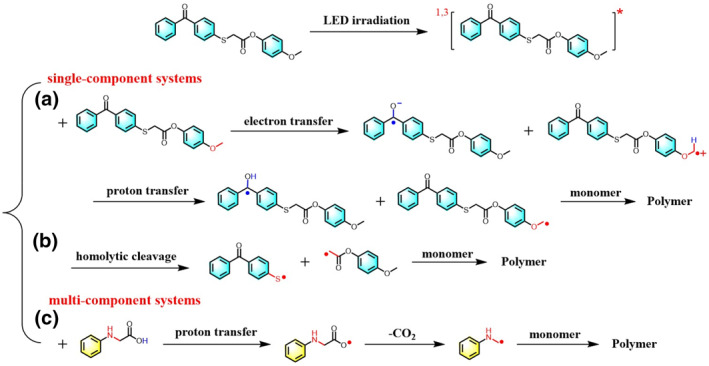
Photochemical reaction mechanism of (a) hydrogen‐capture and (b) splitting in single‐component system, (c) hydrogen‐capture in multi‐component systems. The red part of free radicals indicates their ability to trigger.

The polymerization mechanism of BP‐Sn, as shown in Figure 5, is elucidated based on the preceding findings. Taking BP‐S1 as a representative example. Exposure to 365 nm light irradiation led to the formation of higher energy excited states of BP‐S1. This process facilitates intermolecular electron transfer between BP‐S1 molecules, resulting in the formation of a complex comprising of a radical anion and a radical cation. Following this, proton transfer occurs, yielding an alkoxyl radical and a hydroxymethyl radical (Figure 5a). The alkoxyl radical serves as the initiator for polymerization, whereas the hydroxymethyl radical exhibits relatively stability and remains unreactive due to steric hindrance and electron delocalization, ultimately terminating the reaction via coupling or disproportionation.[^37^, ^38^] In addition, the relatively low bond energy of the C−S bond (approximately 272 kJ mol^−1^)[^25^, ^39^] allows it to cleave under LED light irradiation, producing a methyl radical and a sulfur radical, both of which can initiate polymerization (Figure 5b). The non‐selectivity nature of sulfur radical, along with its diminished sensitivity to oxygen, may contribute the resistance of BP‐Sn to oxygen inhibition.^[40]^ In a two‐component system, the excited BP‐S1 preferentially engages with the co‐initiator NPG through hydrogen abstraction and decarboxylation, leading to the generation of aminoalkyl radicals capable of initiating polymerization (Figure 5c).

### 3.6 Migration stability testing

**FIGURE 6:**
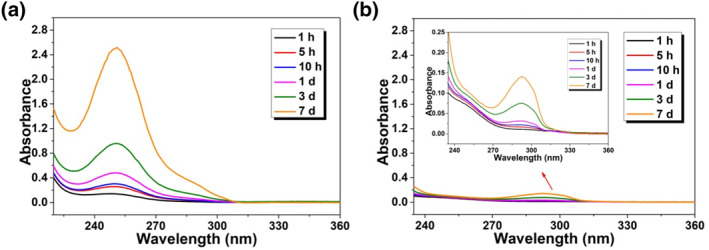
The graph of photoinitiation migration rate. (a) The changes in the absorption spectra of BP immersed in the acetonitrile solution, (b) the changes in the absorption spectra of BP‐S1 under similar conditions in acetonitrile.

The synthesized BP‐Sn and commercially available BP were mixed with monomer resin to prepare a photosensitive liquid to for the purpose of assessing migration stability. This liquid was then cured in a silicone mold and illuminated with an LED lamp. The assessment of migration stability was conducted by analyzing the UV absorption spectra of the cured samples after immersion in anhydrous acetonitrile for a predetermined duration. The comparative analysis of the spectra indicated that the migration rate of the PIs was relatively rapid during the initial hour of immersion, followed by a gradual decrease in the rate of absorbance increase, as the extension of extraction time (Figure 6 and Supporting Information S1: Figure S23). Furthermore, after a 7‐day immersion in anhydrous acetonitrile, the migration mass fractions for BP‐S1, BP‐S2, BP‐S3, BP‐S4, and BP‐S5 were recorded at 4.81%, 6.28%, 3.60%, 4.27%, and 2.32%, respectively (Supporting Information S1: Table S5 and Figure S24), which is one order of magnitude lower than the 38.09% observed for BP. This finding indicates a significant enhancement in the migration stability of the synthesized PIs, as compared to BP. This improvement can be partly attributed to the favorable compatibility between acrylate monomers and BP‐Sn, which promotes a thorough cross‐linking process upon light exposure, thereby enhancing their stability within the polymer matrix.^[41]^ The minimal post‐curing migration of the synthesized BP‐Sn suggests potential for applications in medical devices and food packaging industries.

### 3.7 Curing applications

**FIGURE 7:**
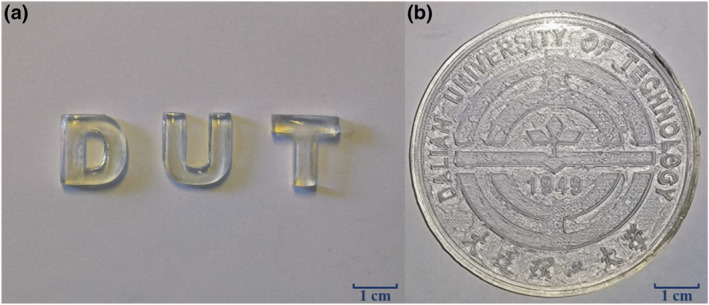
Photocuring model diagram of DUT(a), school emblem (b).

In a conclusive demonstration of the applicability of BP‐Sn in photocurable process, BP‐S5 was chosen as the PI and mixed with the TPGDA monomer with a weight ratio of 1:999. The mixture was agitated to achieve uniformity and then purged with nitrogen. The resultant photosensitive liquid was then poured into custom silicone molds and exposed to LED@365‐nm for 2 min. As shown in Figure 7a, the curing process successfully yielded a distinct and intact pattern of the letters “DUT”, each measuring approximately 10 mm in width. Furthermore, for a more complex emblem pattern of sizes around 70 mm (Figure 7b), the curing process also produced a clear representation of the design elements, with both the pattern and accompanying text easily discernible. The successful curing of both simple and complex patterns, as evidenced by the experiments, underscores the superior moldability of BP‐Sn. These PIs demonstrate the capability to produce detailed and precise forms while ensuring high‐fidelity replication across various sizes and designs. Such characteristics are particularly valuable in applications requiring high‐resolution and intricate detailing, including the fabrication of microfluidic devices, precision components, and fine art pieces.

## 4 CONCLUSION

In summary, we have successfully developed a series of sulfur‐containing BP‐based PIs (BP‐Sn), which improve the solubility and initiation efficiency of BP through the deliberate incorporation of ester groups and hydrogen‐donating substituents. These PIs exhibit exceptional photopolymerization performance at low concentrations (0.1 wt%), achieving nearly 95% conversion efficiency and a photoinitiation rate exceeding 20% s^−1^ for TPGDA, without the need for additional co‐initiators. Furthermore, they exhibit excellent thermal stability, allowing for prolonged storage at ambient temperature without degradation. The low migration rate (less than 5%) of these PIs suggests their suitability for sensitive applications such as medical devices and food packaging, where material purity and safety are paramount. Additionally, the versatility of their chemical structures may facilitate the development of tailored variants to meet specific industrial needs, such as high‐resolution 3D printing or advanced electronic materials. Exploring these avenues will not only maximize the functionality of these PIs but also promote the progress in the fields of materials science. Overall, this work lays a solid foundation for the development of next‐generation photopolymerization materials, with potential to inspire new research directions and practical applications across various fields.

## CONFLICT OF INTEREST STATEMENT

The authors declare no conflict of interests.

## ETHICS STATEMENT

No animal or human experiments were involved in this study.

## Supporting information

Supporting Information S1

## Data Availability

Data openly available in a public repository.
