# Supplementary material for: A novel sulfur‐containing photoinitiator based on benzophenone derivatives for rapid photopolymerization
Source: Smart Mol. 2025 Mar 28;4(2):e20240065. doi: 10.1002/smo.20240065 (PMC13317665; doi:10.1002/smo.20240065)
Supplement: Supplementary file 1 — Supporting Information S1 [file SMO2-4-e20240065-s001.docx]

**Supporting Information**

**A Novel Sulfur-Containing Photoinitiator Based on Benzophenone Derivatives for Rapid Photopolymerization**

Yu Li, Wenzheng Li, Saihe Yang, Lu Li, Shikun Song, Yuting Tang, Han Han, Zhenyue Duan, Yangyang Xin, Jiangli Fan; Pengzhong Chen* and Xiaojun Peng

CONTENT

[1 Synthesis of compounds 1](#_Toc184723038)

[2 Molecular structure characterization 5](#_Toc184723039)

[3 Thermal stability analysis 14](#_Toc184723040)

[4 UV-Vis absorption spectra 15](#_Toc184723041)

[5 Steady-state photodegradation spectra 16](#_Toc184723042)

[6 Calculations of the free energy changes of electron transfer 17](#_Toc184723043)

[7 Single excited state energy 18](#_Toc184723044)

[8 Photopolymerization 19](#_Toc184723045)

[9 Electron paramagnetic resonance testing 20](#_Toc184723046)

[10 Migration stability testing 21](#_Toc184723047)

# Synthesis of compounds


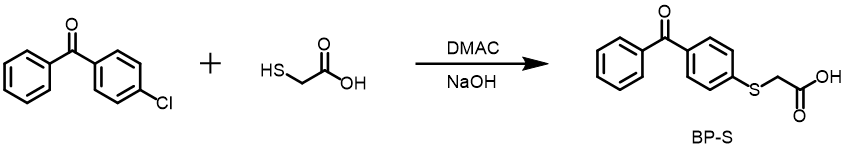


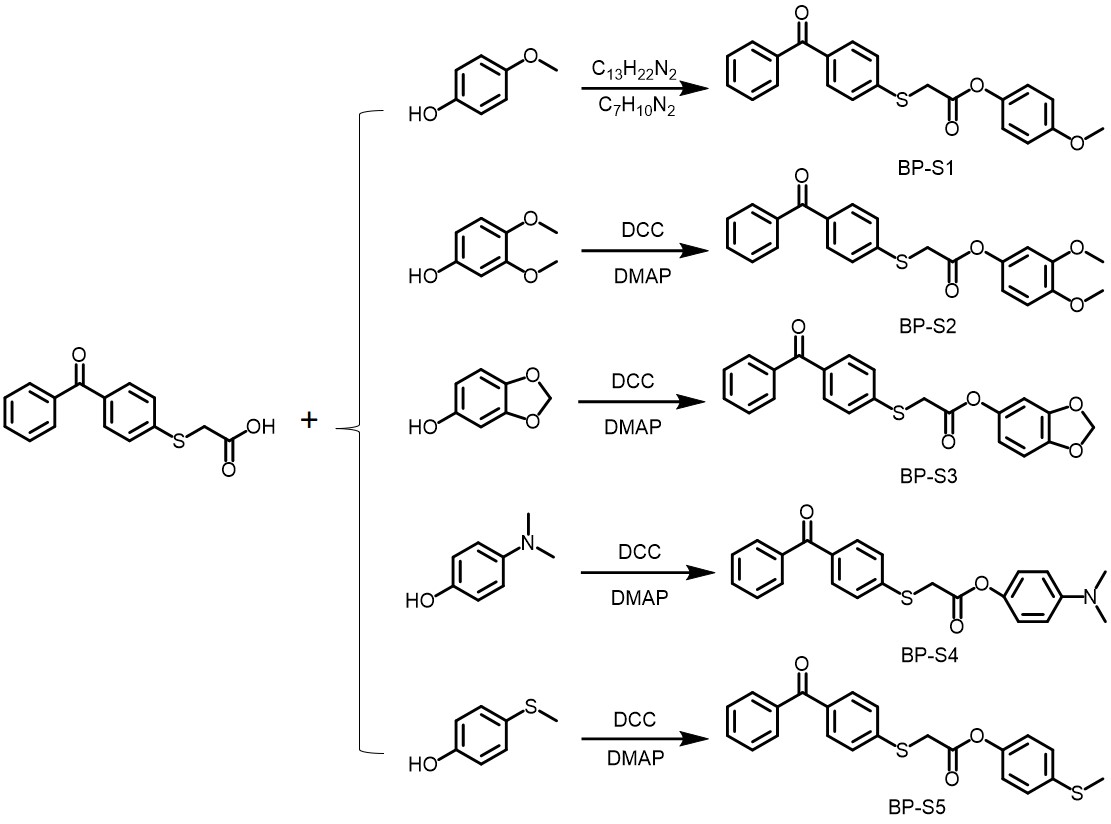


Scheme S1: The synthetic pathway for the compounds.

**Synthesis of 2-((4-Benzoylphenyl) thio) acetic acid (BP-S)**

1. In a 100 mL round-bottom flask fitted with a stirrer, thermometer, and reflux condenser, 4-chlorobenzophenone (3.25 g, 15.00 mmol) and mercaptoacetic acid (1.66 g, 1.25 mL, 18.00 mmol) were introduced. The mixture was dissolved in 8 mL of *N, N*-dimethylacetamide (DMAC), to which solid sodium hydroxide (1.20 g) was subsequently added. The reaction mixture was subjected to reflux (105-110 °C) until the solution turned orange-yellow.

2. The progress of the reaction was monitored using thin-layer chromatography (TLC), with samples being collected at regular intervals. Upon confirmation of the consumption of 4-chlorobenzophenone, which occurred approximately 8 hours, the reaction was terminated. The reaction mixture was subsequently transferred into 50 mL of water, and the aqueous phase was extracted with three portions of 10 mL toluene. The PH of the aqueous phase was adjusted to a range of 3 to 4 using concentrated hydrochloric acid, resulting in the precipitation of a white solid. The solid was subjected to filtration, followed by washing and drying to yield a crude product (2.95 g) with a yield of 72.30%.

3. Recrystallization from dichloromethane yielded 2.53 g of a white crystalline product, with a yield of 61.94%.

^1^H NMR (600 MHz, DMSO-*d*_6_) δ 12.93 (s, 1H), 7.72 (d, J = 7.7 Hz, 2H), 7.68 (t, J = 8.0 Hz, 3H), 7.57 (t, J = 7.5 Hz, 2H), 7.47 – 7.44 (m, 2H), 3.97 (s, 2H).

HRMS (ESI, m/z): C_15_H_11_O_3_S [M-H]^-^ calculated for 271.0429，observed: 271.0427.

**Synthesis of 4-Methoxyphenyl 2-((4-Benzoylphenyl) thio) acetate (BP-S1)**

1. In a 100 mL round-bottom flask, BP-S (272.32 mg, 1.00 mmol), 4-methoxyphenol (136.55 mg, 1.10 mmol), *N, N*'-dicyclohexylcarbodiimide (DCC) (412.67 mg, 2.00 mmol), and 4-dimethylaminopyridine (DMAP) (24.43 mg, 0.20 mmol) were combined. The reactants were dissolved in 15 mL of anhydrous dichloromethane and stirred at room temperature to initiate the reaction.

2. The progress of the reaction was monitored using thin-layer chromatography (TLC). Upon completion, the reaction mixture was transferred to a separatory funnel and washed with water three times. The organic phase was collected, and anhydrous sodium sulfate was added to remove water, followed by filtration and rotary evaporation to yield the crude product.

3. Purification was performed via precipitation. The crude product was dispersed in ethanol, subjected to stirring and heating for a duration of 30 minutes, followed by filtration, washing with water, and rinsed with ethanol. The product was dried under vacuum to yield the purified product (103.40 mg) with a yield of 27.23%.

^1^H NMR (600 MHz, CDCl_3_) δ 7.79 – 7.76 (m, 4H), 7.61 – 7.57 (m, 1H), 7.53 – 7.47 (m, 4H), 6.98 – 6.94 (m, 2H), 6.89 – 6.85 (m, 2H), 3.95 (s, 2H), 3.79 (s, 3H).

^13^C NMR (101 MHz, DMSO) δ 195.34, 168.71, 157.54, 144.20, 142.31, 137.61, 134.52, 133.06, 130.88, 129.94, 129.06, 126.94, 122.79, 115.00, 55.90, 34.06.

HRMS (ESI, m/z): C_22_H_18_O_4_SNa [M+Na]^+^ calculated for 401.0818, observed: 401.0816.

**Synthesis of 3,4-Dimethoxyphenyl 2-((4-Benzoylphenyl) thio) acetate (BP-S2)**

1. In a 100 mL round-bottom flask, BP-S (272.32 mg, 1.00 mmol), 3,4-dimethoxyphenol (169.58 mg, 1.10 mmol), *N, N*'-dicyclohexylcarbodiimide (DCC) (412.67 mg, 2.00 mmol), and 4-dimethylaminopyridine (DMAP) (24.43 mg, 0.20 mmol) were introduced. The mixture was dissolved in 15 mL of anhydrous dichloromethane and stirred at room temperature to initiate the reaction.

2. The reaction progress was monitored using thin-layer chromatography (TLC). After completion, the reaction mixture was transferred to a separatory funnel and washed with water three times. The organic layer was collected and subsequently dried using an appropriate quantity of anhydrous sodium sulfate. Following this, the mixture was filtered, and the solvent was removed by rotary evaporation to obtain the crude product.

3. Purification was performed via precipitation. The crude product was dispersed in ethanol, subjected to stirring and heating for a duration of 30 minutes, followed by filtration, washing with water, and rinsed with ethanol. The product was dried under vacuum to yield the purified product (75.60 mg) with a yield of 18.51%.

^1^H NMR (600 MHz, CDCl_3_) δ 7.80 – 7.76 (m, 4H), 7.59 (t, J = 7.4 Hz, 1H), 7.53 (d, J = 8.5 Hz, 2H), 7.48 (t, J = 7.7 Hz, 2H), 6.82 (d, J = 8.7 Hz, 1H), 6.60 (dd, J = 8.7, 2.7 Hz, 1H), 6.56 (d, J = 2.6 Hz, 1H), 3.96 (s, 2H), 3.86 (s, 3H), 3.83 (s, 3H).

^13^C NMR (101 MHz, DMSO) δ 200.10, 173.35, 154.37, 151.98, 149.11, 147.02, 142.35, 139.32, 137.82, 135.62, 134.69, 133.82, 131.84, 117.92, 117.03, 111.19, 61.02, 60.87, 38.91.

HRMS (ESI, m/z): C_23_H_20_O_5_SNa [M+Na]^+^ calculated for 431.0924, observed: 431.0931.

**Synthesis of benzo[*d*] [1,3] dioxol-5-yl 2-((4-Benzoylphenyl) thio) acetate (BP-S3)**

1. In a 100 mL round-bottom flask, 3,4-methylenedioxyphenol (151.93 mg, 1.10 mmol), BP-S (272.32 mg, 1.00 mmol), *N, N*'-dicyclohexylcarbodiimide (DCC) (412.67 mg, 2.00 mmol), and 4-dimethylaminopyridine (DMAP) (24.43 mg, 0.20 mmol) were introduced. The mixture was dissolved in 15 mL of anhydrous dichloromethane and stirred at room temperature to initiate the reaction.

2. The reaction progress was monitored using thin-layer chromatography (TLC). After completion, the reaction mixture was poured into a separatory funnel and washed with water three times. The organic layer was collected, dried with an appropriate quantity of anhydrous sodium sulfate. Following this, the mixture was filtered, and the solvent was removed by rotary evaporation to obtain the crude product.

3. Purification was performed via precipitation. The crude product was dispersed in ethanol, subjected to stirring and heating for a duration of 30 minutes, followed by filtration, washing with water, and rinsed with ethanol. The product was subjected to vacuum to yield the purified product (87.60 mg) with a yield of 22.32%.

^1^H NMR (600 MHz, CDCl_3_) δ 7.79 – 7.76 (m, 4H), 7.59 (d, J = 7.4 Hz, 1H), 7.52 – 7.47 (m, 4H), 6.75 (d, J = 8.4 Hz, 1H), 6.55 (d, J = 2.3 Hz, 1H), 6.48 (dd, J = 8.4, 2.4 Hz, 1H), 5.98 (s, 2H), 3.93 (s, 2H).

^13^C NMR (101 MHz, DMSO-*d_6_*) δ 195.35, 168.63, 148.13, 145.63, 145.07, 142.25, 137.61, 134.54, 133.05, 130.88, 129.94, 129.06, 126.99, 114.37, 108.48, 104.01, 102.28, 34.08.

HRMS (ESI, m/z): C_22_H_16_O_5_SNa [M+Na]^+^ calculated for 415.0611, observed: 415.0613.

**Synthesis of 4-(Dimethylamino) phenyl 2-((4-Benzoylphenyl) thio) acetate (BP-S4)**

1. In a 100 mL round-bottom flask, BP-S (272.32 mg, 1.00 mmol), 4-dimethylaminophenol (150.90 mg, 1.10 mmol), *N, N*'-dicyclohexylcarbodiimide (DCC) (412.67 mg, 2.00 mmol), and 4-dimethylaminopyridine (DMAP) (24.43 mg, 0.20 mmol) were combined. The mixture was dissolved in 15 mL of anhydrous dichloromethane and stirred at room temperature to initiate the reaction.

2. The reaction progress was monitored using thin-layer chromatography (TLC). Upon completion of the reaction, the reaction mixture was poured into a separatory funnel and washed with water three times. The organic phase was collected, dried using anhydrous sodium sulfate, filtered, and the solvent was subsequently removed through rotary evaporation to yield the crude product.

3. Purification was performed via precipitation. The crude product was dispersed in ethanol, subjected to stirring and heating for a duration of 30 minutes, followed by filtration, washing with water, and rinsed with ethanol. The product was dried under vacuum to yield the purified product (109.50 mg) with a yield of 27.97%.

^1^H NMR (600 MHz, DMSO-*d*_6_) δ 7.72 (d, J = 7.3 Hz, 4H), 7.68 (t, J = 7.3 Hz, 1H), 7.58 – 7.53 (m, 4H), 6.92 – 6.85 (m, 2H), 6.72 – 6.68 (m, 2H), 4.31 (s, 2H), 2.86 (s, 6H).

^13^C NMR (101 MHz, DMSO-*d_6_*) δ 195.34, 168.88, 149.11, 142.40, 141.41, 137.62, 134.49, 133.05, 130.86, 129.94, 129.06, 126.93, 122.06, 113.23, 40.88, 34.08.

HRMS (ESI, m/z): C_23_H_21_NO_3_SNa [M+Na]^+^ calculated for 414.1134, observed: 414.1133.

**Synthesis of 4-(Methylthio) phenyl 2-((4-Benzoylphenyl) thio) acetate (BP-S5)**

1. In a 100 mL round-bottom flask, BP-S (272.32 mg, 1.00 mmol), 4-methylthiophenol (154.22 mg, 1.10 mmol), *N, N*'-dicyclohexylcarbodiimide (DCC) (412.67 mg, 2.00 mmol), and 4-dimethylaminopyridine (DMAP) (24.43 mg, 0.20 mmol) were introduced. The mixture was dissolved in 15 mL of anhydrous dichloromethane and stirred at room temperature to initiate the reaction.

2. The reaction progress was monitored using thin-layer chromatography (TLC). After completion, the reaction mixture was poured into a separatory funnel and washed with water three times. The organic layer was collected, dried with an appropriate quantity of anhydrous sodium sulfate. Following this, the mixture was filtered, and the solvent was removed by rotary evaporation to obtain the crude product.

3. Purification was performed via precipitation. The crude product was dispersed in ethanol, subjected to stirring and heating for a duration of 30 minutes, followed by filtration, washing with water, and rinsed with ethanol. The product was dried under vacuum to yield the purified product (105.20 mg) with a yield of 26.67%.

^1^H NMR (600 MHz, DMSO-*d*_6_) δ 7.72 (dd, J = 5.2, 4.2 Hz, 4H), 7.67 (t, J = 7.0 Hz, 1H), 7.56 (t, J = 7.4 Hz, 4H), 7.30 (d, J = 8.5 Hz, 2H), 7.05 (d, J = 8.6 Hz, 2H), 4.35 (s, 2H), 2.46 (s, 3H).

^13^C NMR (101 MHz, DMSO-*d*_6_) δ 195.34, 168.49, 148.25, 142.23, 137.61, 136.24, 134.55, 133.06, 130.89, 129.94, 129.06, 127.63, 126.97, 122.59, 34.10, 15.56.

HRMS (ESI, m/z): C_22_H_18_O_3_S_2_Na [M+Na]^+^ calculated for 417.0589, observed: 417.0587.

# Molecular structure characterization


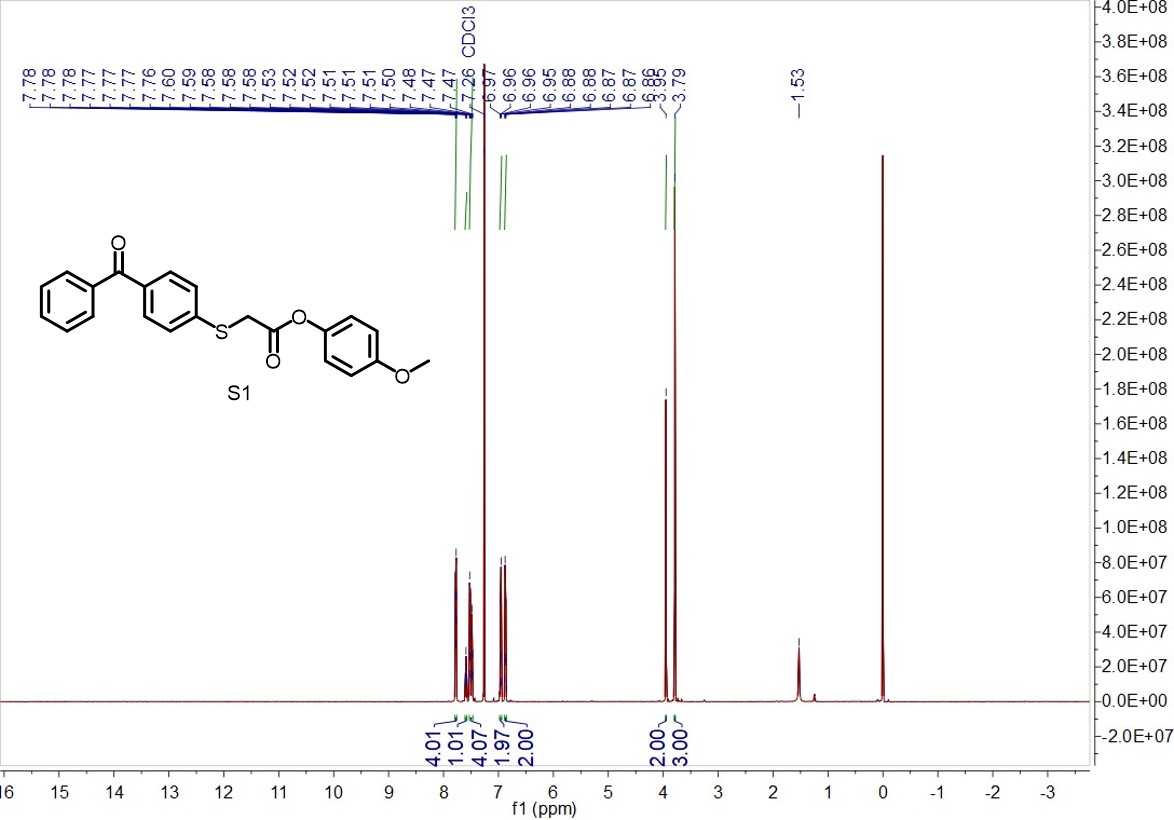


**Figure S1.** ^1^H NMR spectrum of **BP-S1** in CDCl_3_.


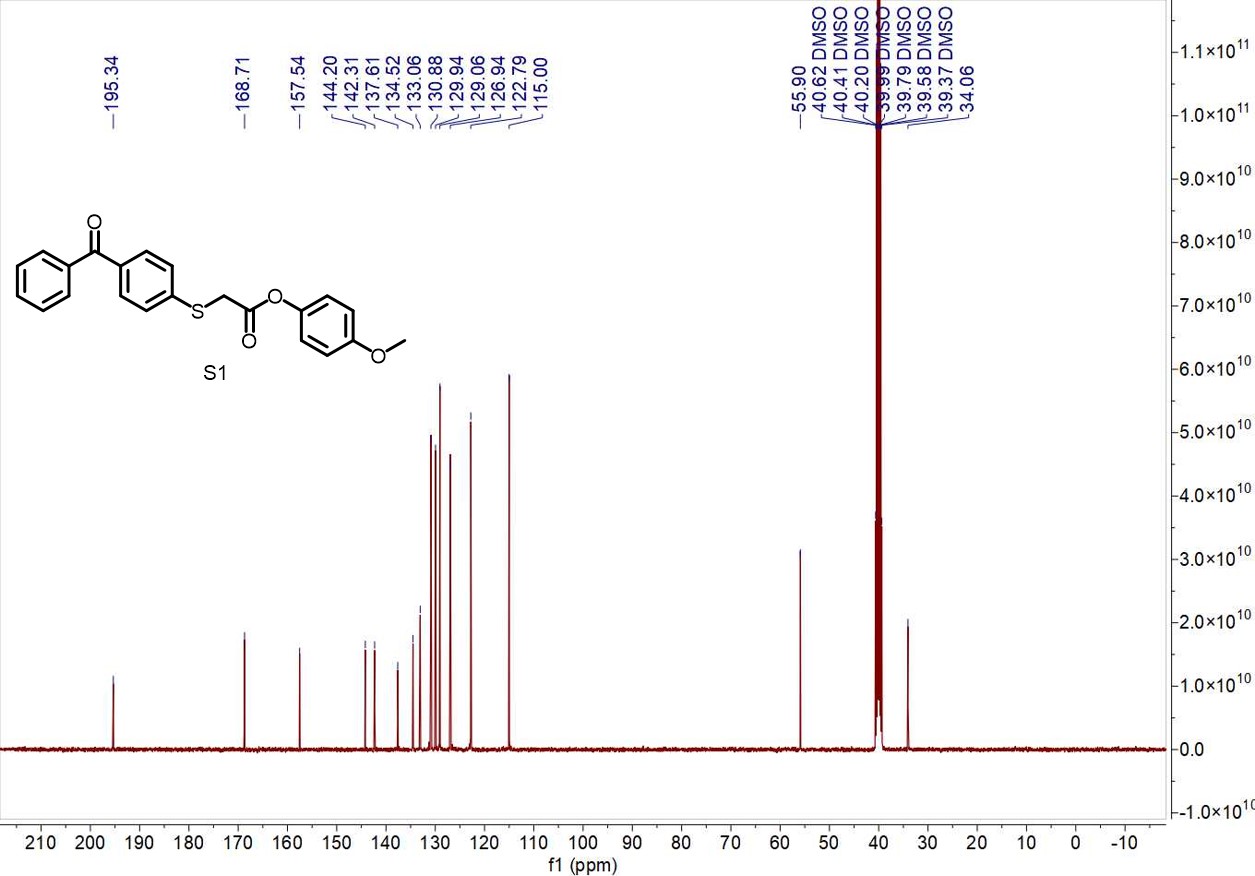


**Figure S2.** ^13^C NMR spectra of **BP-S1** in DMSO.


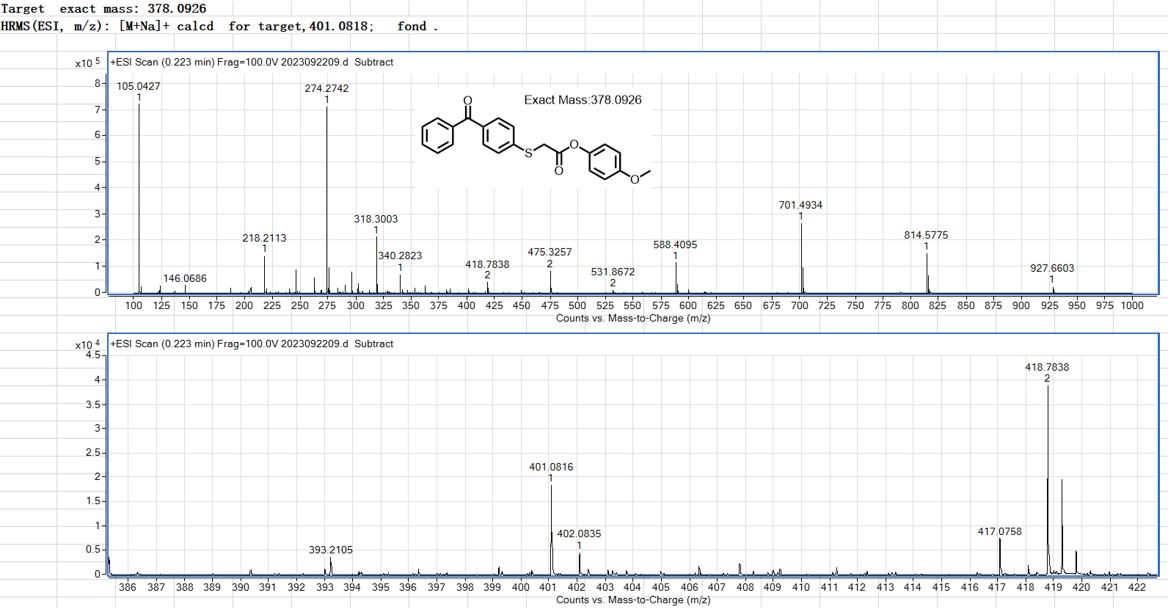


**Figure S3.** HRMS spectra of **BP-S1**.


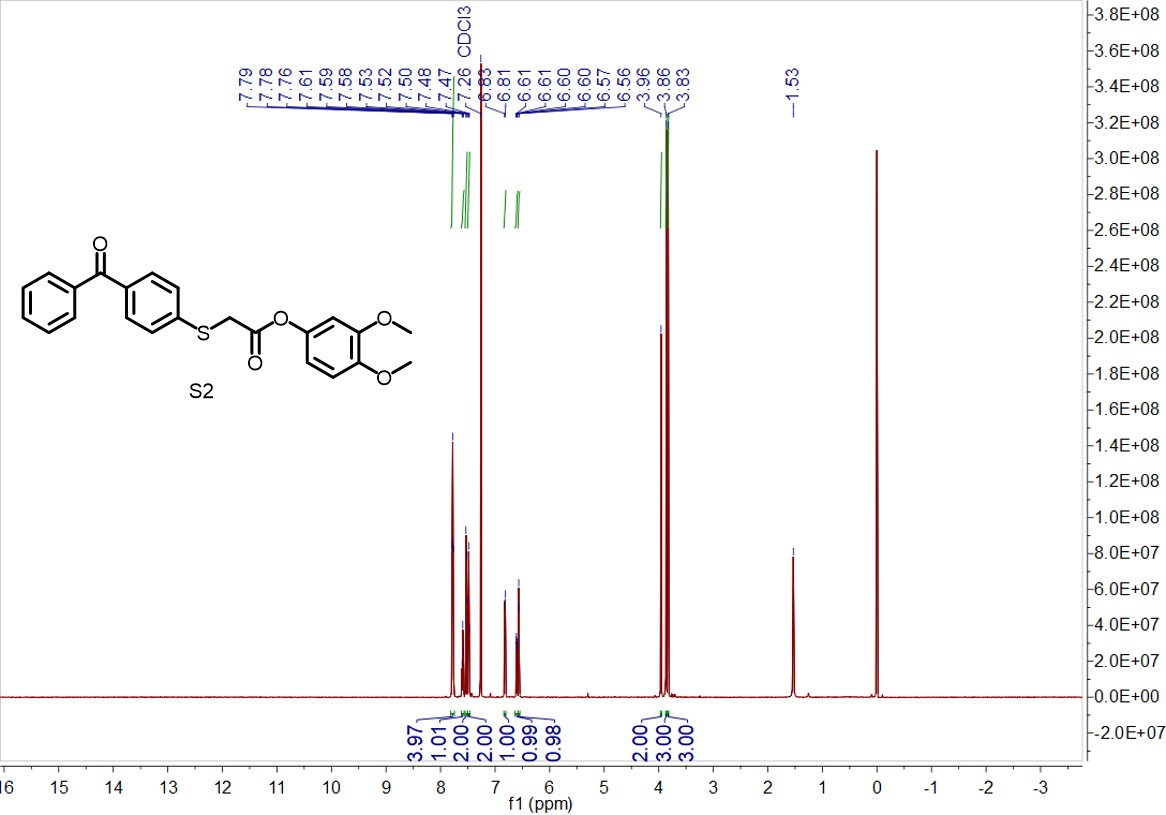


**Figure S4.** ^1^H NMR spectra of **BP-S2** in CDCl_3_.


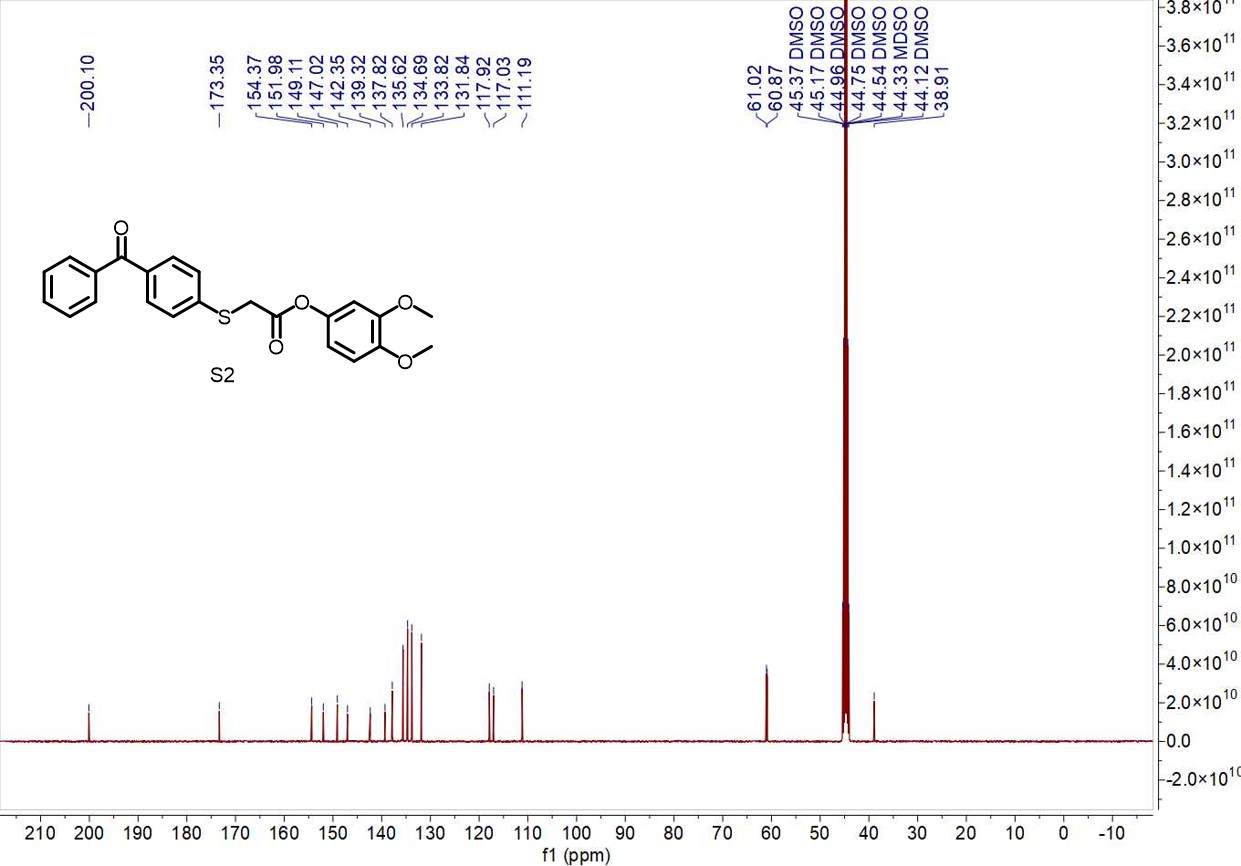


**Figure S5.** ^13^C NMR spectra of **BP-S2** in DMSO.


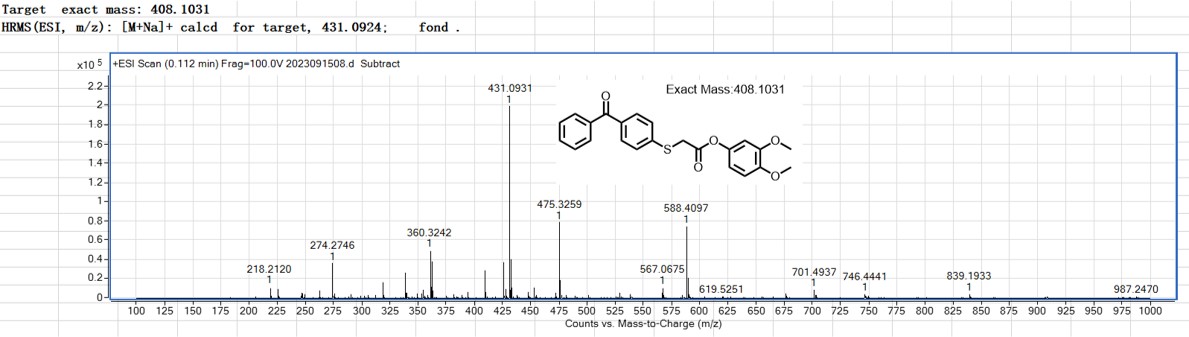


**Figure S6.** HRMS spectra of **BP-S2**.


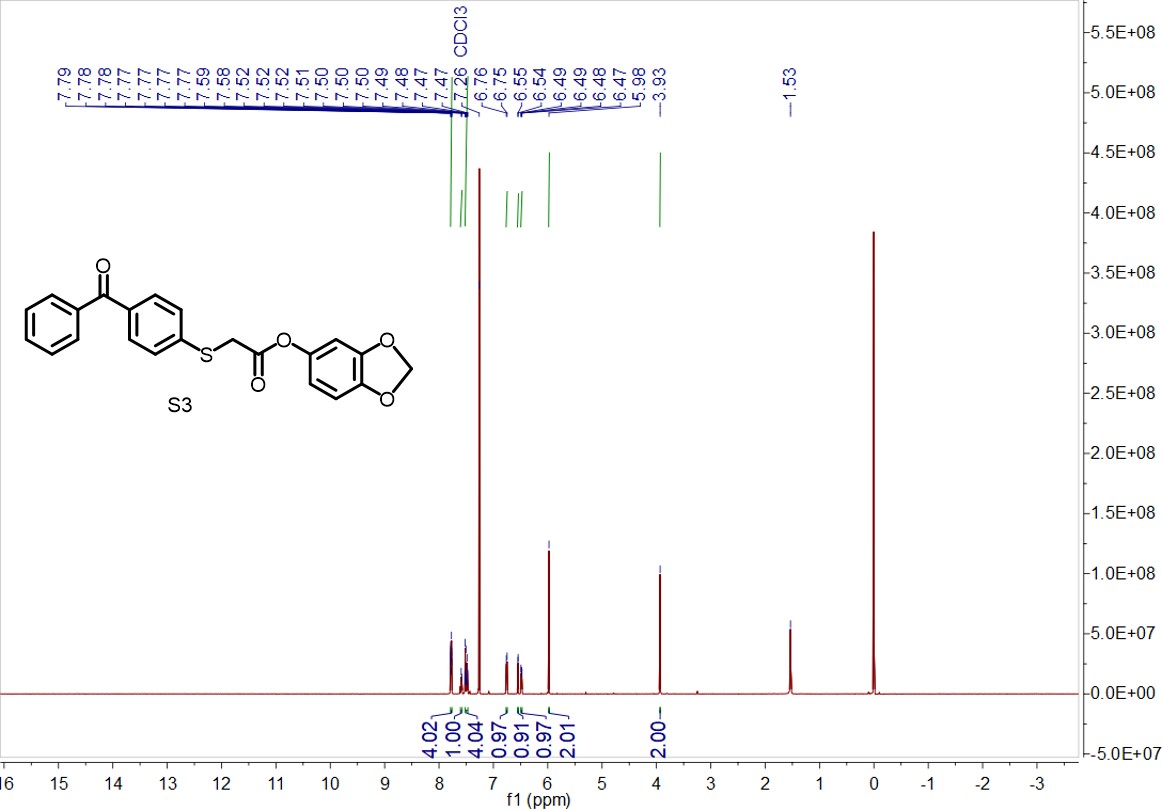


**Figure S7.** ^1^H NMR spectra of **BP-S3** in CDCl_3_.


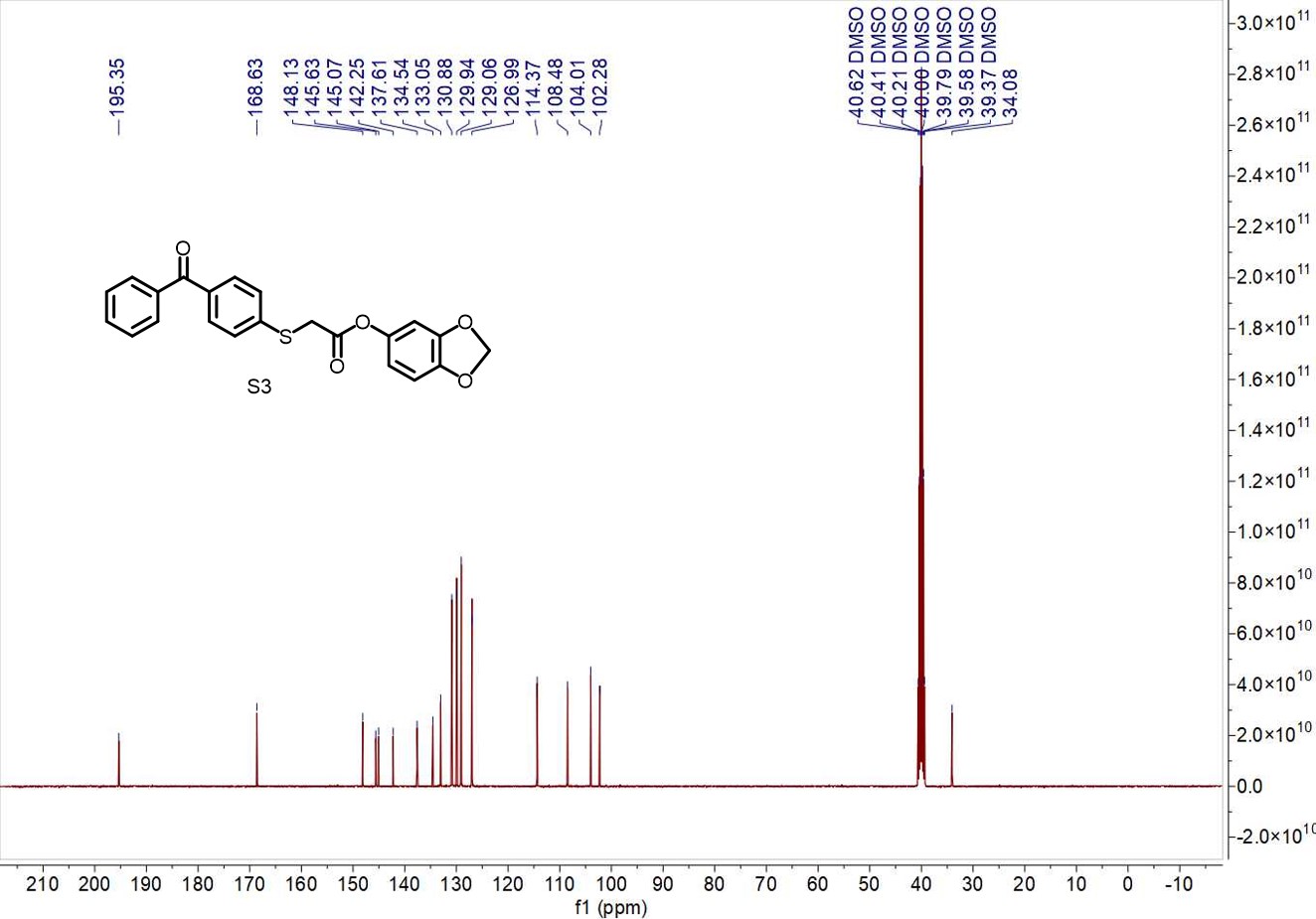


**Figure S8.** ^13^C NMR spectra of **BP-S3** in DMSO.


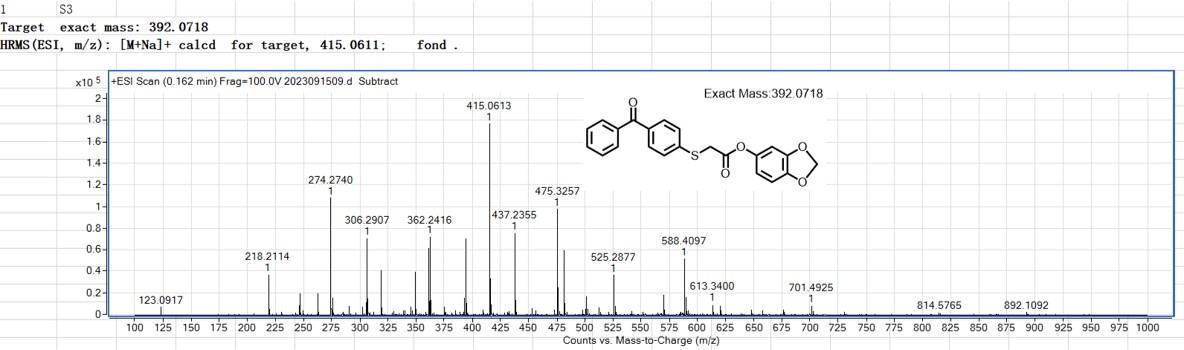


**Figure S9.** HRMS spectra of **BP-S3**.


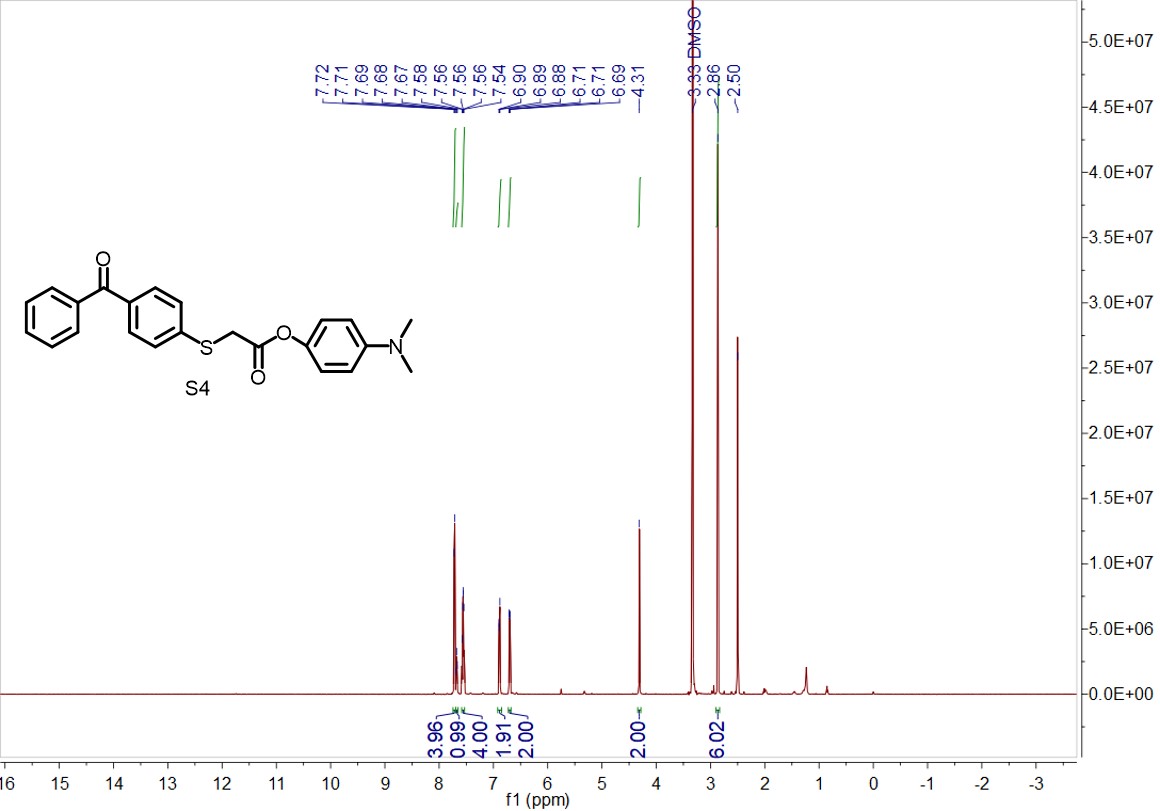


**Figure S10.** ^1^H NMR spectra of **BP-S4** in DMSO.


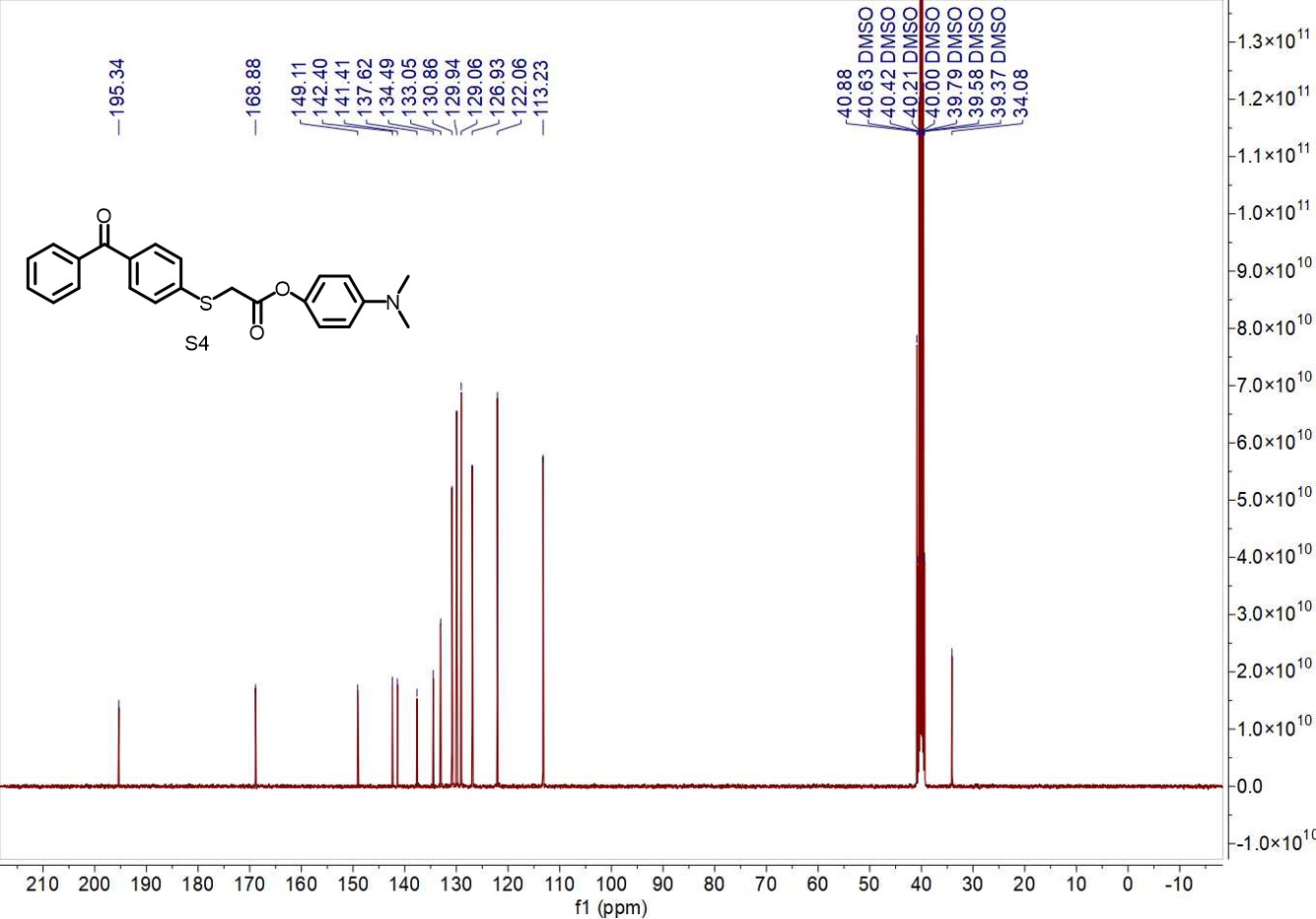


**Figure S11.** ^13^C NMR spectra of **BP-S4** in DMSO.


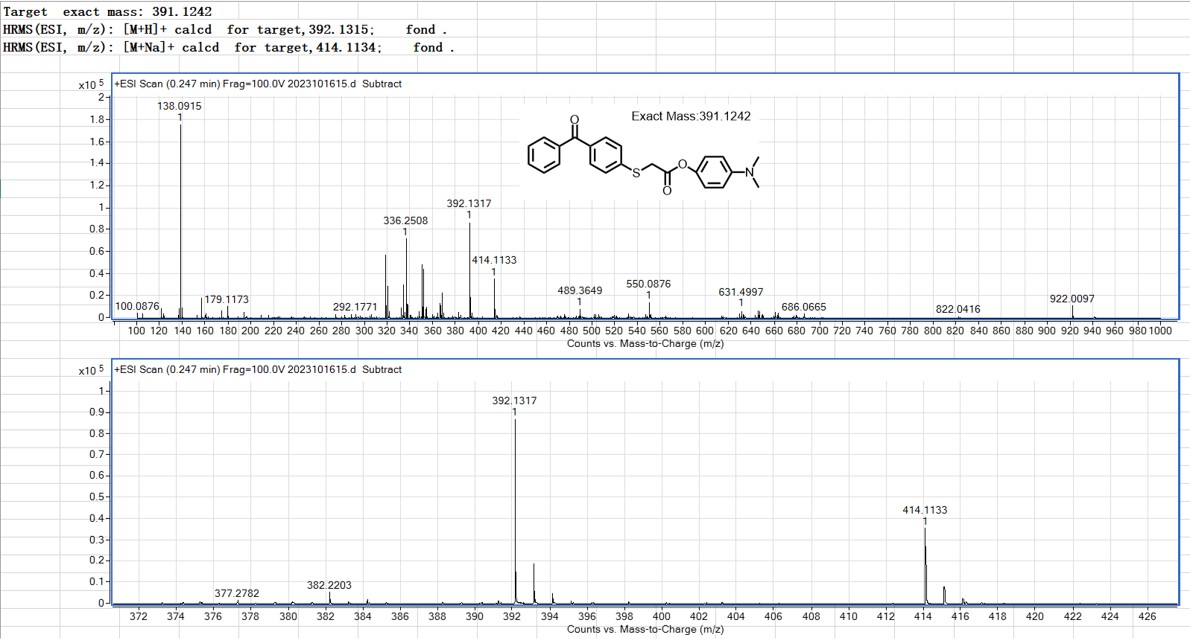


**Figure S12.** HRMS spectra of **BP-S4**.


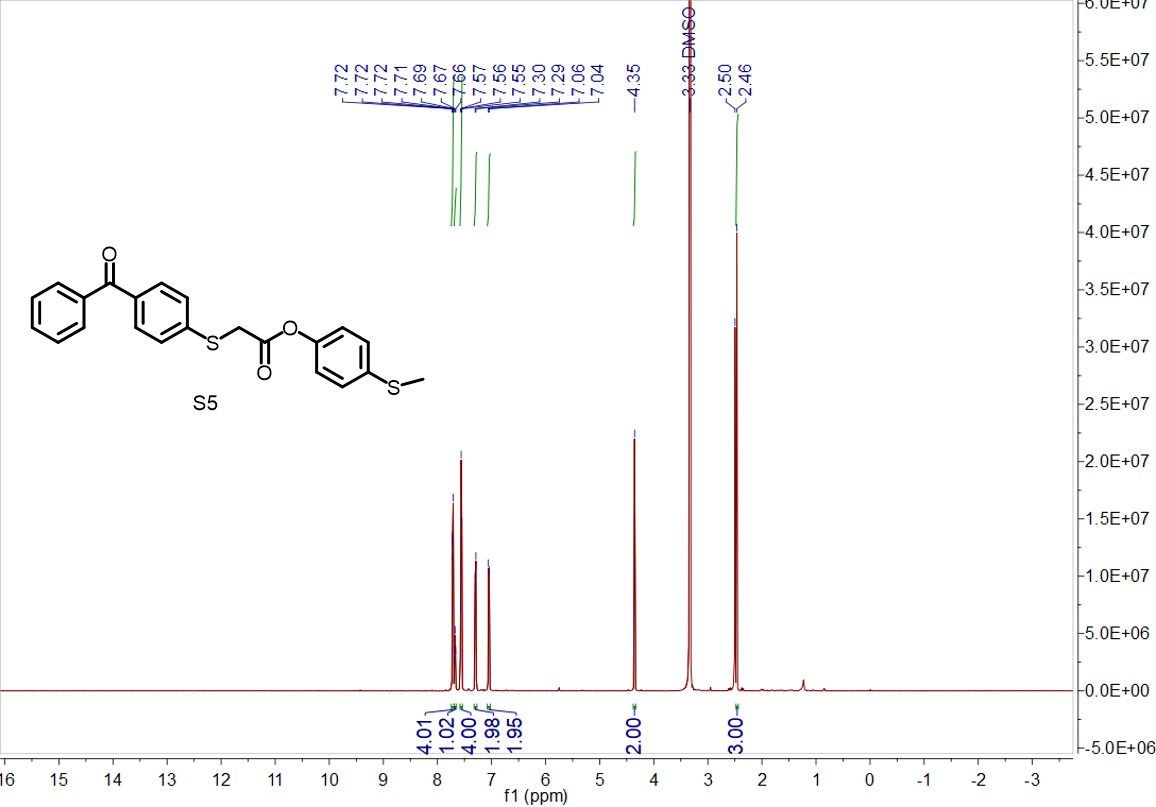


**Figure S13.** ^1^H NMR spectra of **BP-S5** in DMSO.


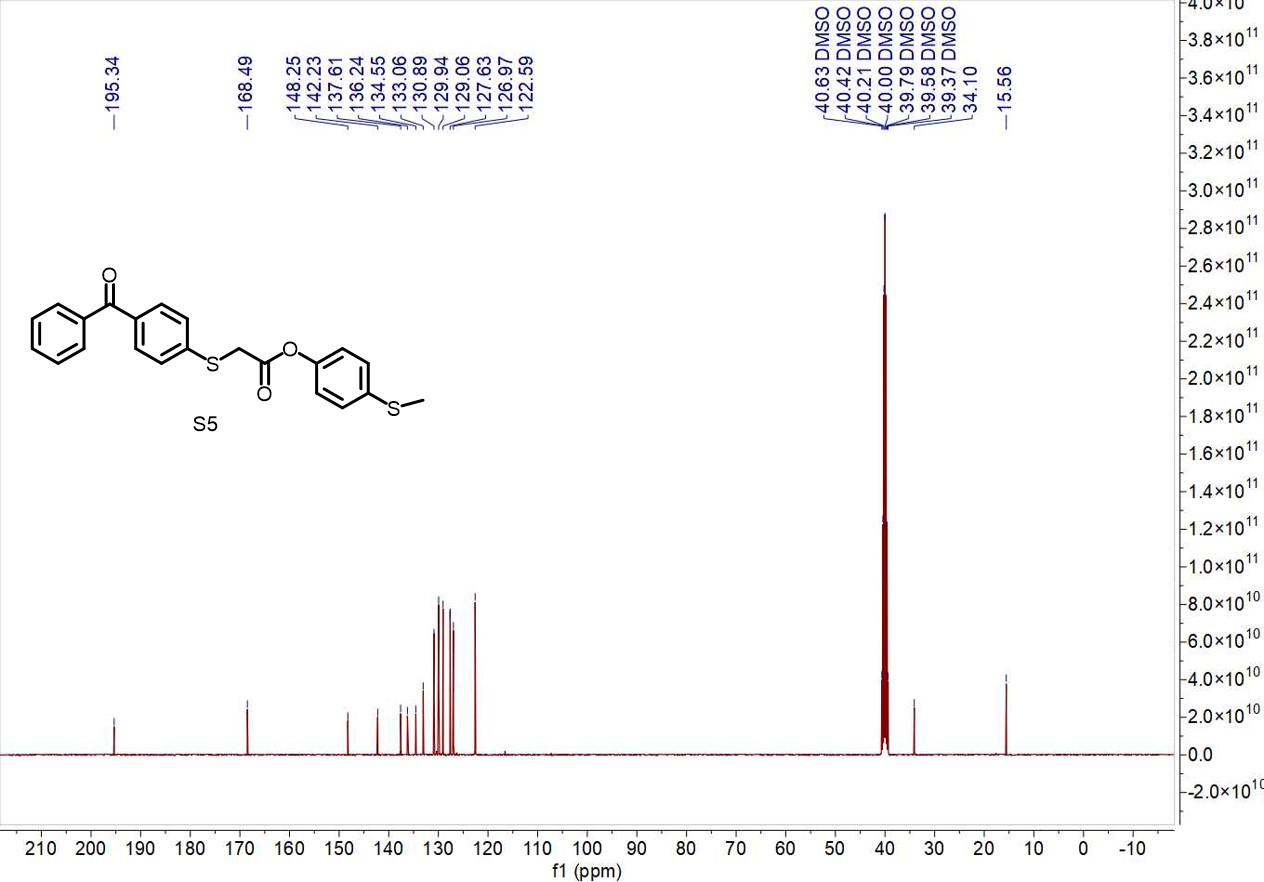


**Figure S14.** ^13^C NMR spectra of **BP-S5** in DMSO.


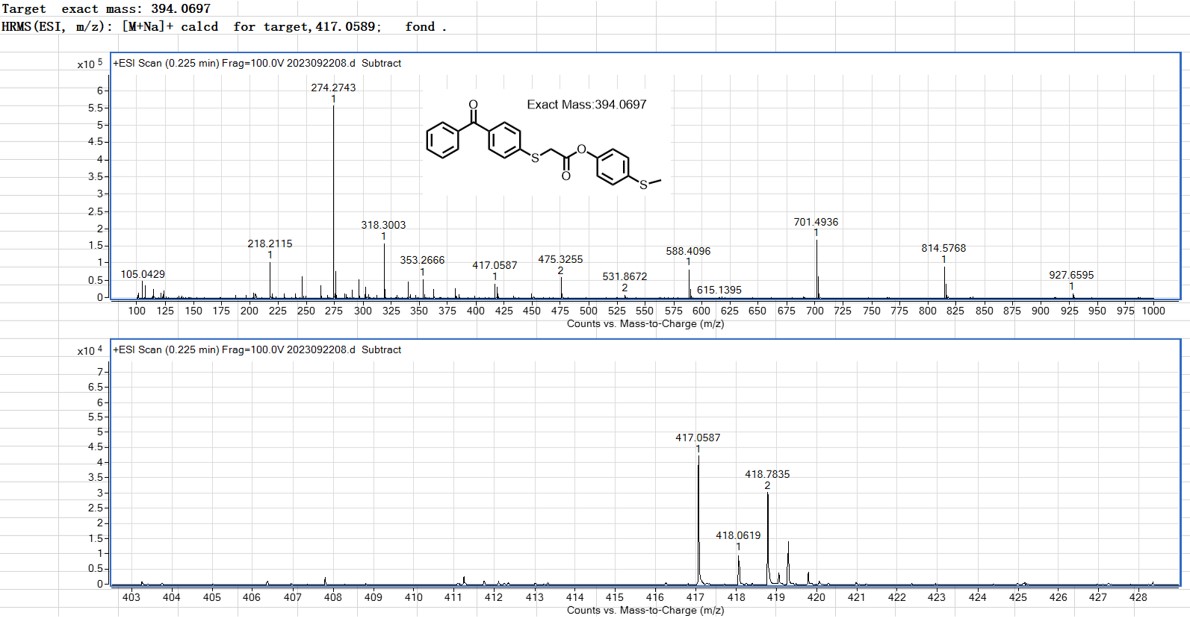


**Figure S15.** HRMS spectra of **BP-S5**.

# Thermal stability analysis

**Table S1**. Thermogravimetric analysis data table for BP-Sn.

| **PI** | Thermal weight loss rate at 250 °C | T_10%_ (°C) | T_50%_ (°C) | T_max_ (°C) |
| --- | --- | --- | --- | --- |
| **BP-S1** | 0.82% | 311.17 | 353.33 | 465.33 |
| **BP-S2** | 0.97% | 312.17 | 348.00 | 431.50 |
| **BP-S3** | 1.87% | 303.33 | 352.67 | 443.50 |
| **BP-S4** | 1.01% | 307.67 | 347.33 | 420.67 |
| **BP-S5** | 1.83% | 304.00 | 353.33 | 465.50 |


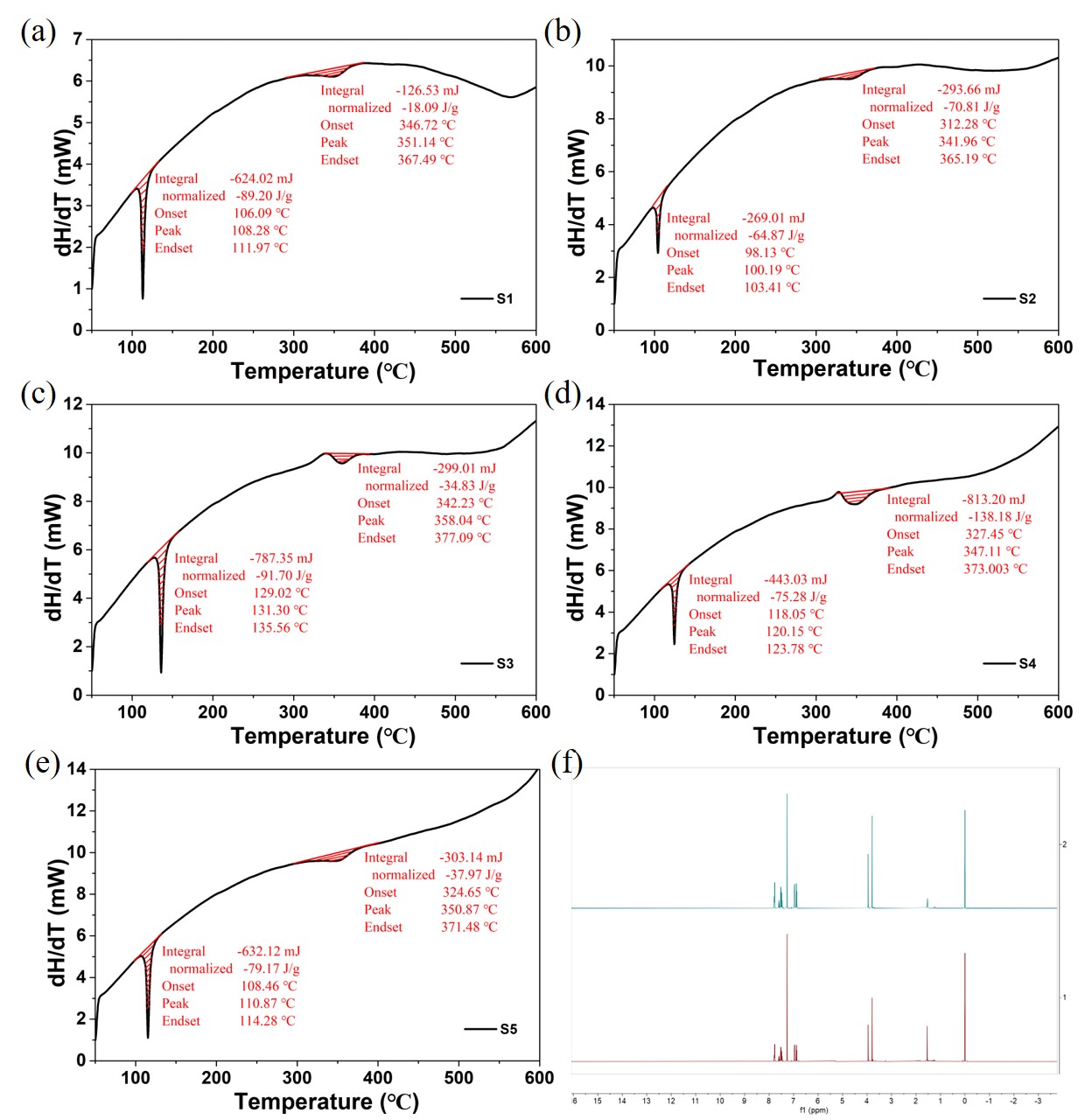


**Figure S16**. Differential scanning calorimetry (DSC) analysis of **BP-S1** (a), **BP-S2** (b), **BP-S3** (c), **BP-S4** (d), **BP-S5** (e) and comparison of ^1^H NMR spectrum of **BP-S1** (the above is one year later)in CDCl_3_ (f).

# UV-Vis absorption spectra


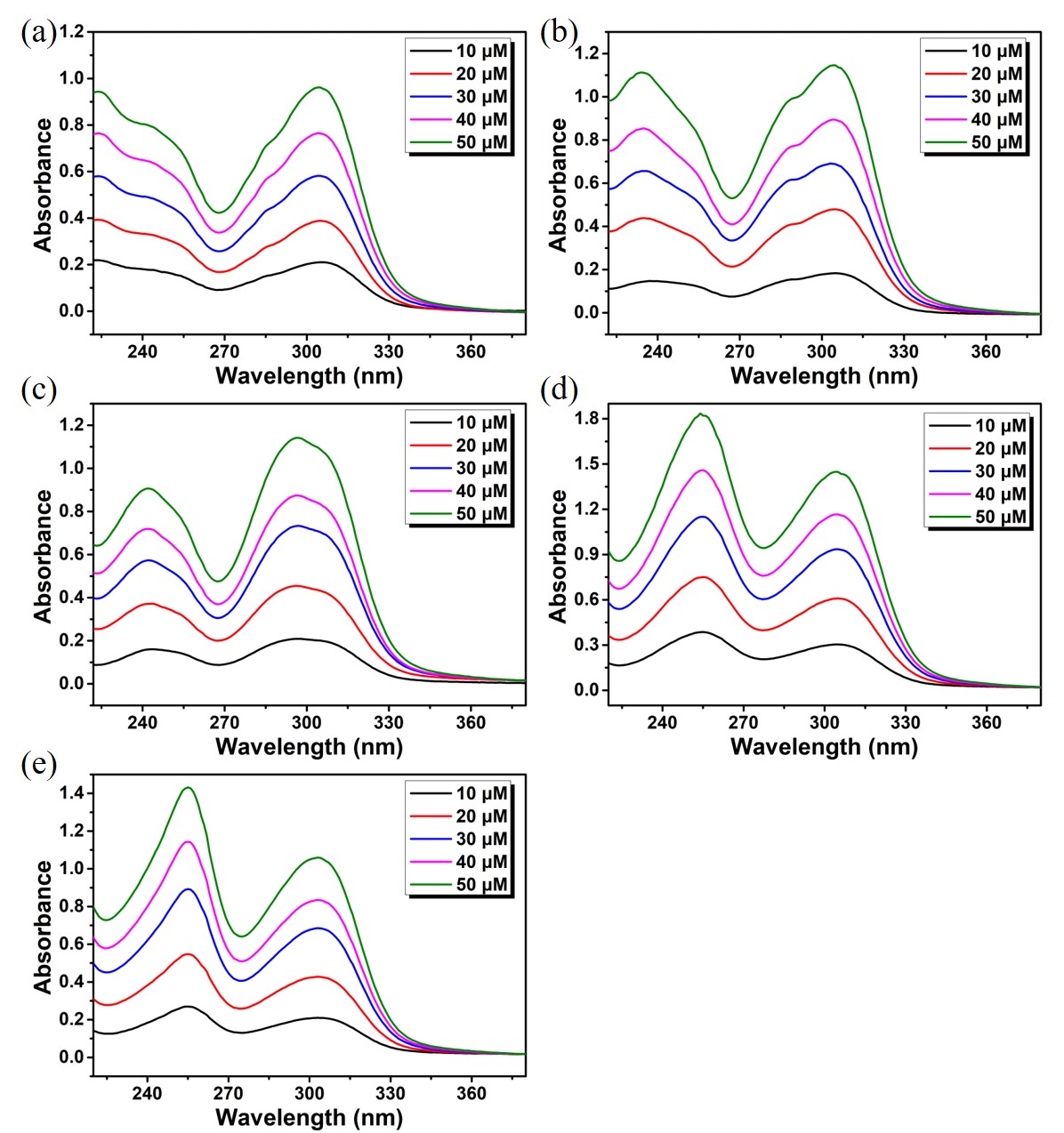


**Figure S17**. The UV-Vis absorption spectra of **BP-S1** (a), **BP-S2** (b), **BP-S3** (c), **BP-S4** (d), and **BP-S5** (e) in acetonitrile solution at 298 K.

**Table S2**. Photophysical property parameters of BP-Sn.

| PI | λ (nm) | ε_max_ (M^-1^∙cm^-1^) | ε_365_ (M^-1^∙cm^-1^) | λ_ex_ (nm) | QY (%) |
| --- | --- | --- | --- | --- | --- |
| BP-S1 | 224, 304 | 18830 | 179 | 355 | 0.7% |
| BP-S2 | 234, 304 | 23520 | 300 | 383 | 0.5% |
| BP-S3 | 243, 297 | 22840 | 438 | 354 | 0.8% |
| BP-S4 | 254, 304 | 36080 | 337 | 381 | 0.8% |
| BP-S5 | 255, 303 | 29200 | 202 | 351 | 0.8% |

# Steady-state photodegradation spectra


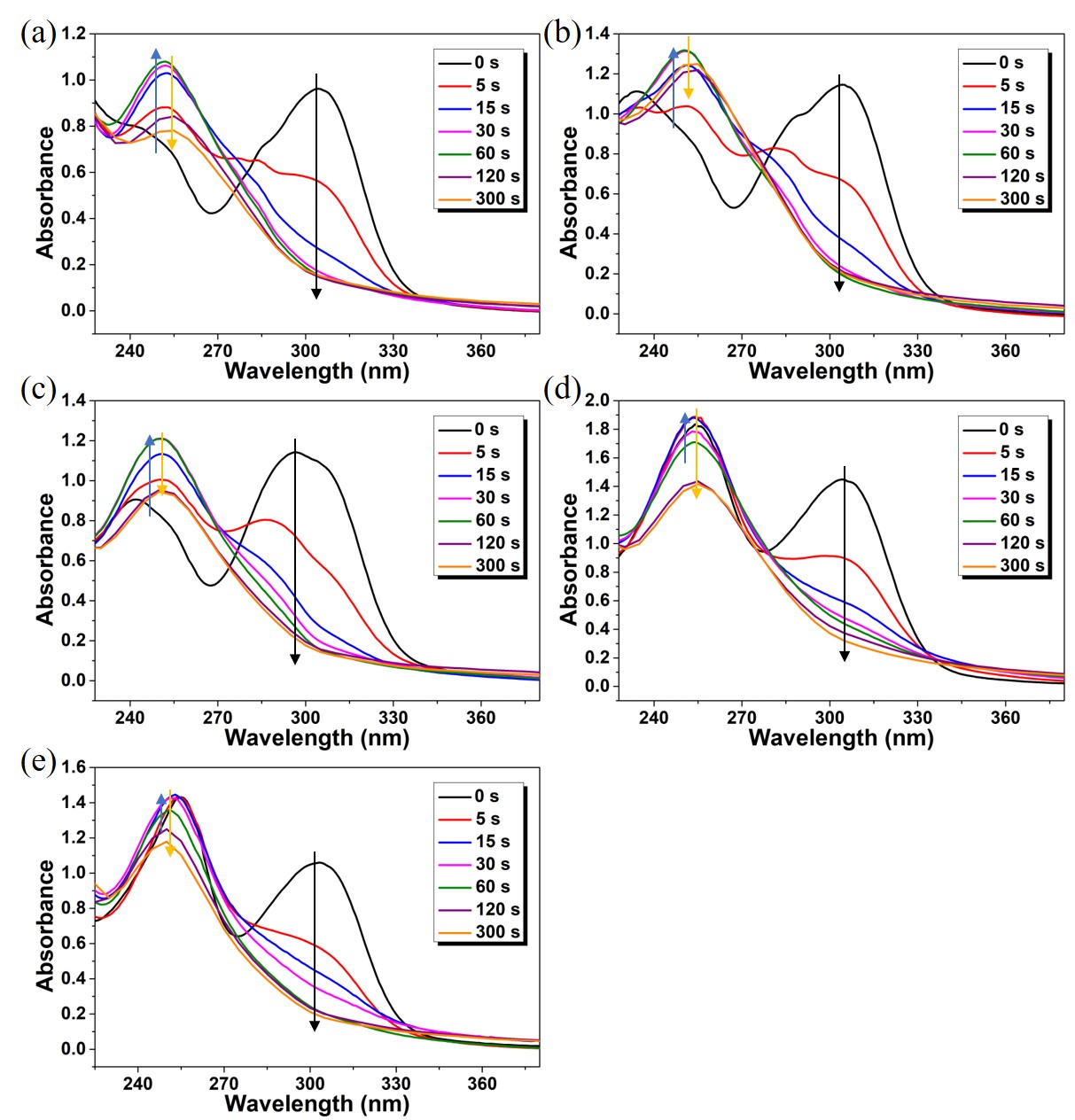


**Figure S18**. Steady-state photodegradation spectra of **BP-S1** (a), **BP-S2** (b), **BP-S3** (c), **BP-S4** (d), and **BP-S5** (e) in acetonitrile solution, c ≈ 5ⅹ10^-5^ mol ∙L^-1^, 298 K.

**Table S3**. Photodegradation rates of five photoinitiators.

| **PI** | **A_0/_A** | **R_d_/mol**∙**L-^1^**∙**s^-1^** |
| --- | --- | --- |
| BP-S1 | 0.16 | 4.41×10^-6^ |
| BP-S2 | 0.17 | 4.16×10^-6^ |
| BP-S3 | 0.18 | 3.75×10^-6^ |
| BP-S4 | 0.22 | 3.76×10^-6^ |
| BP-S5 | 0.17 | 4.63×10^-6^ |

# Calculations of the free energy changes of electron transfer


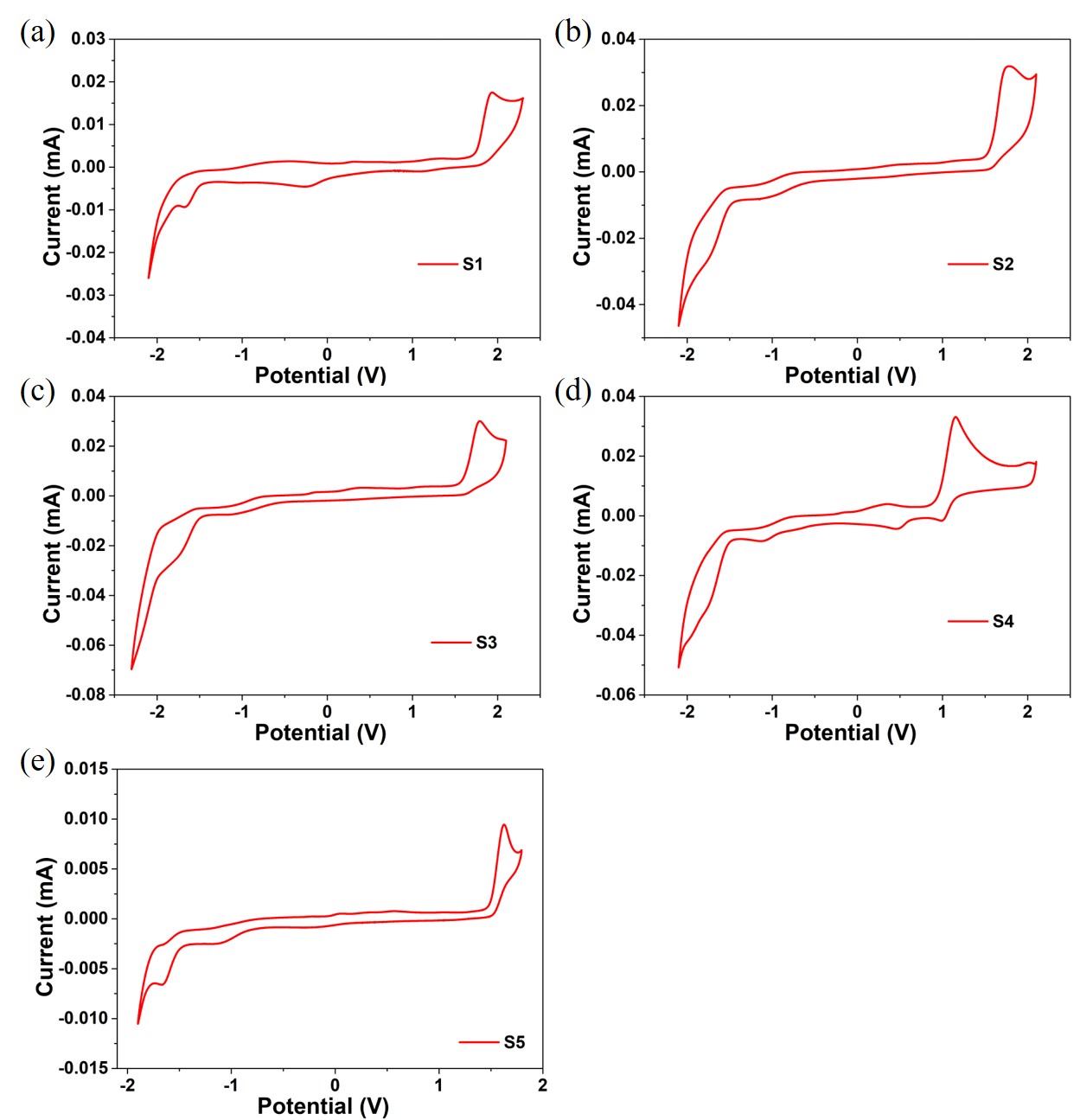


**Figure S19**. Cyclic voltammetry spectra of **BP-S1** (a), **BP-S2** (b), **BP-S3** (c), **BP-S4** (d) and **BP-S5** (e) in anhydrous acetonitrile.

# Single excited state energy


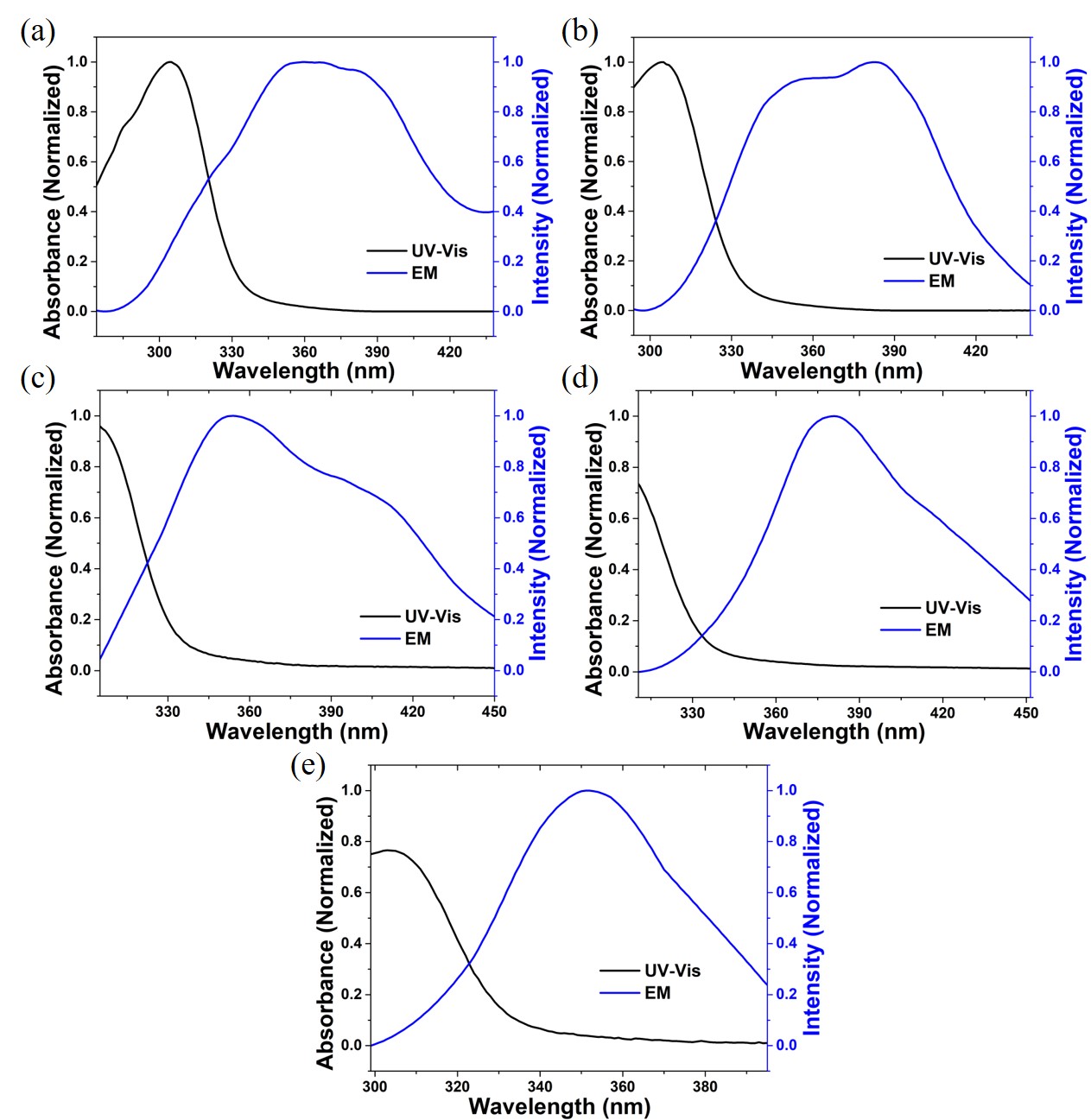


**Figure S20**. UV absorption (left) and fluorescence emission (right) spectra of **BP-S1** (a), **BP-S2** (b), **BP-S3** (c), **BP-S4** (d) and **BP-S5** (e) in anhydrous acetonitrile.

# Photopolymerization


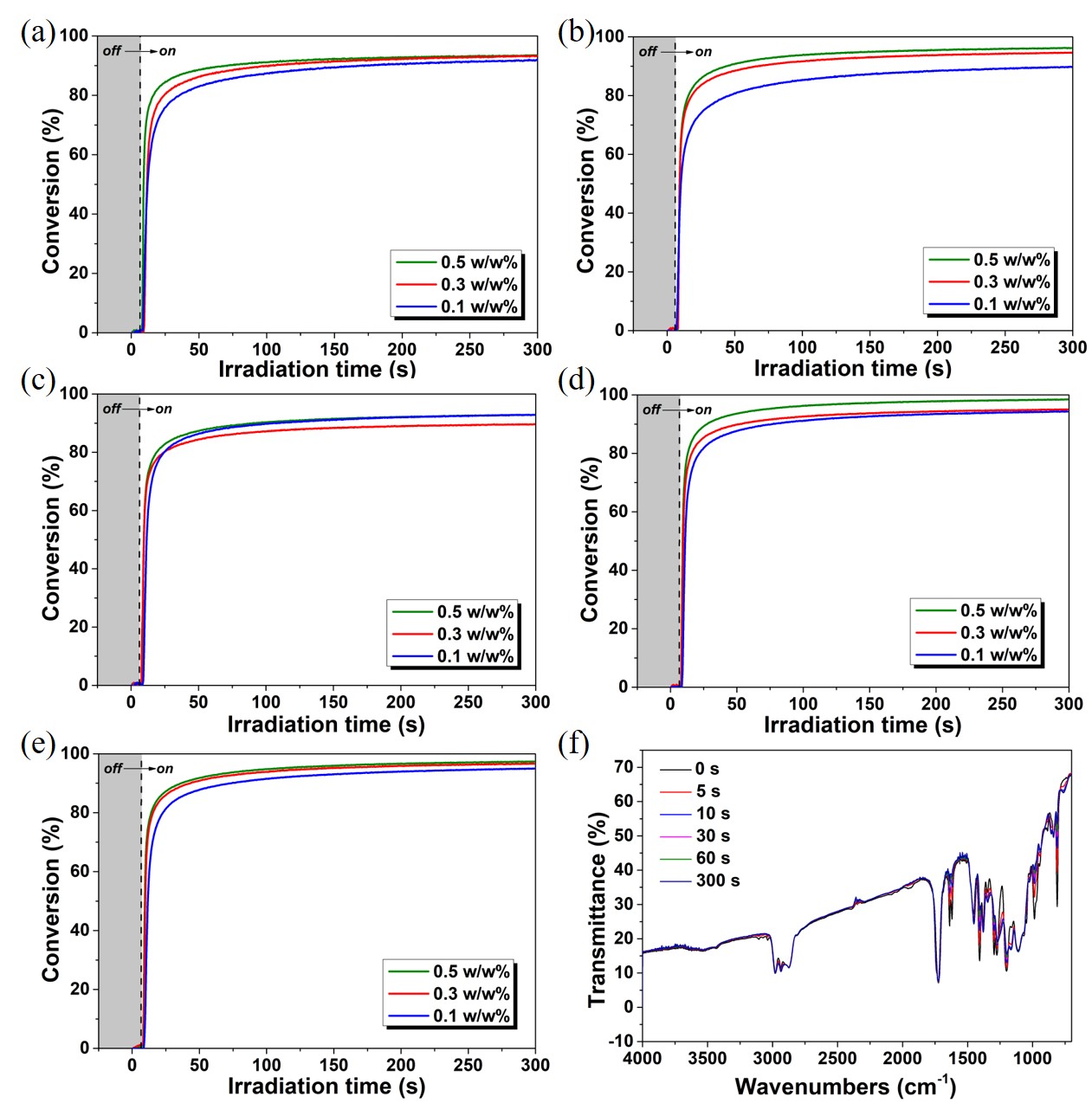


**Figure S21**. Dynamics curves of monomer TPGDA polymerization induced by different concentrations of **BP-S1** (a), **BP-S2** (b), **BP-S3** (c), **BP-S4** (d), **BP-S5** (e) and the raw FTIR spectra from the photopolymerization experiments of **BP-S1**(f). The conversion rate of double bonds is calculated from the change in peak area at 793-830 cm^-1^ in the raw FTIR spectra.

**Table S4**. Kinetic data table for monomer TPGDA polymerization initiated by different concentrations of BP-Sn.

| PI | 0.5 w/w% | 0.3 w/w% | 0.1 w/w% |
| --- | --- | --- | --- |
| BP-S1 | 93.48% | 93.26% | 91.93% |
| BP-S2 | 96.16% | 94.54% | 89.69% |
| BP-S3 | 92.82% | 89.59% | 92.84% |
| BP-S4 | 98.49% | 95.04% | 94.32% |
| BP-S5 | 97.36% | 96.67% | 94.94% |

# Electron paramagnetic resonance testing


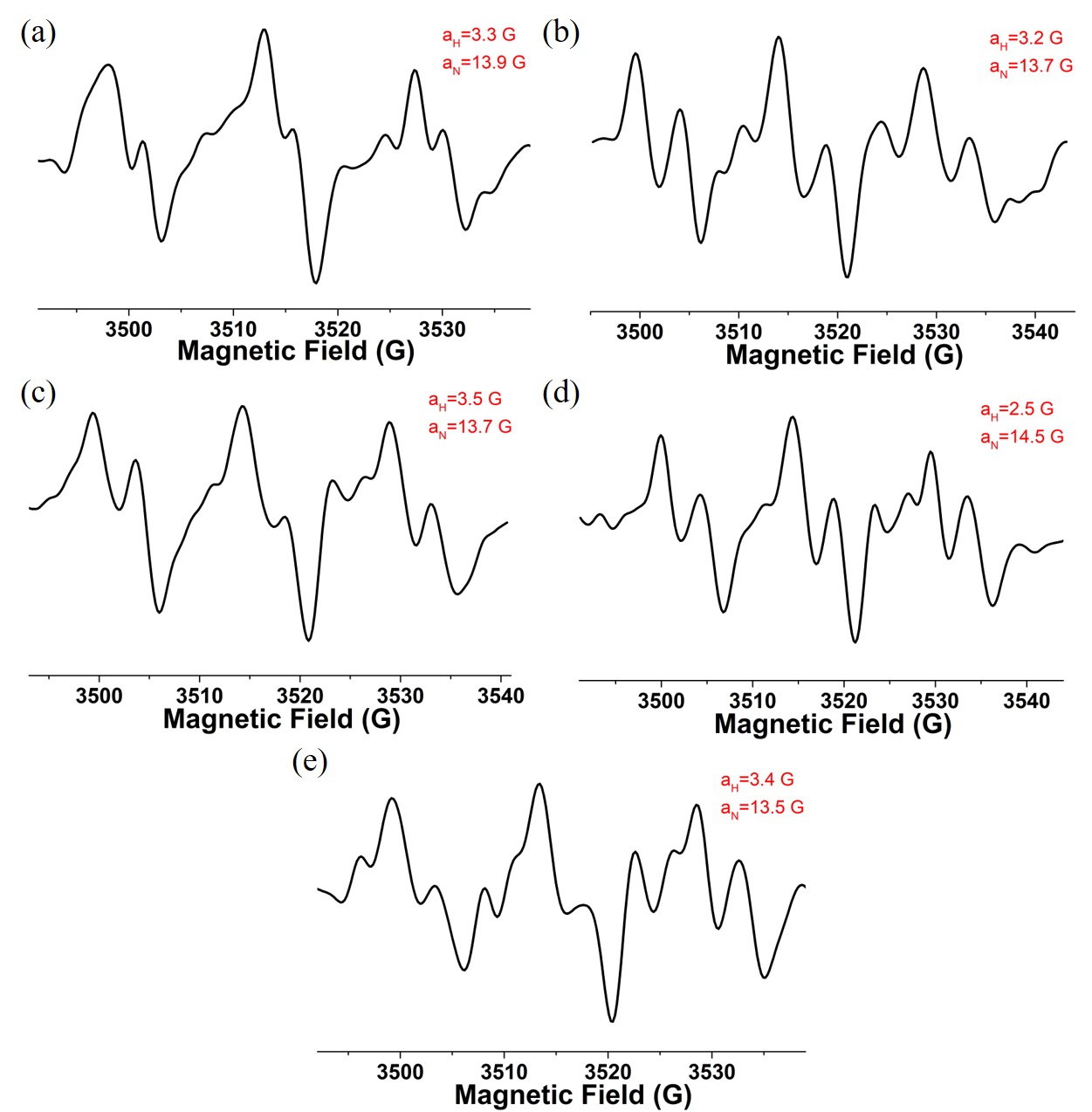


**Figure S22**. ESR spectrogram of **BP-S1** (a), **BP-S2** (b), **BP-S3** (c), **BP-S4** (d) and **BP-S5** (e).

# Migration stability testing


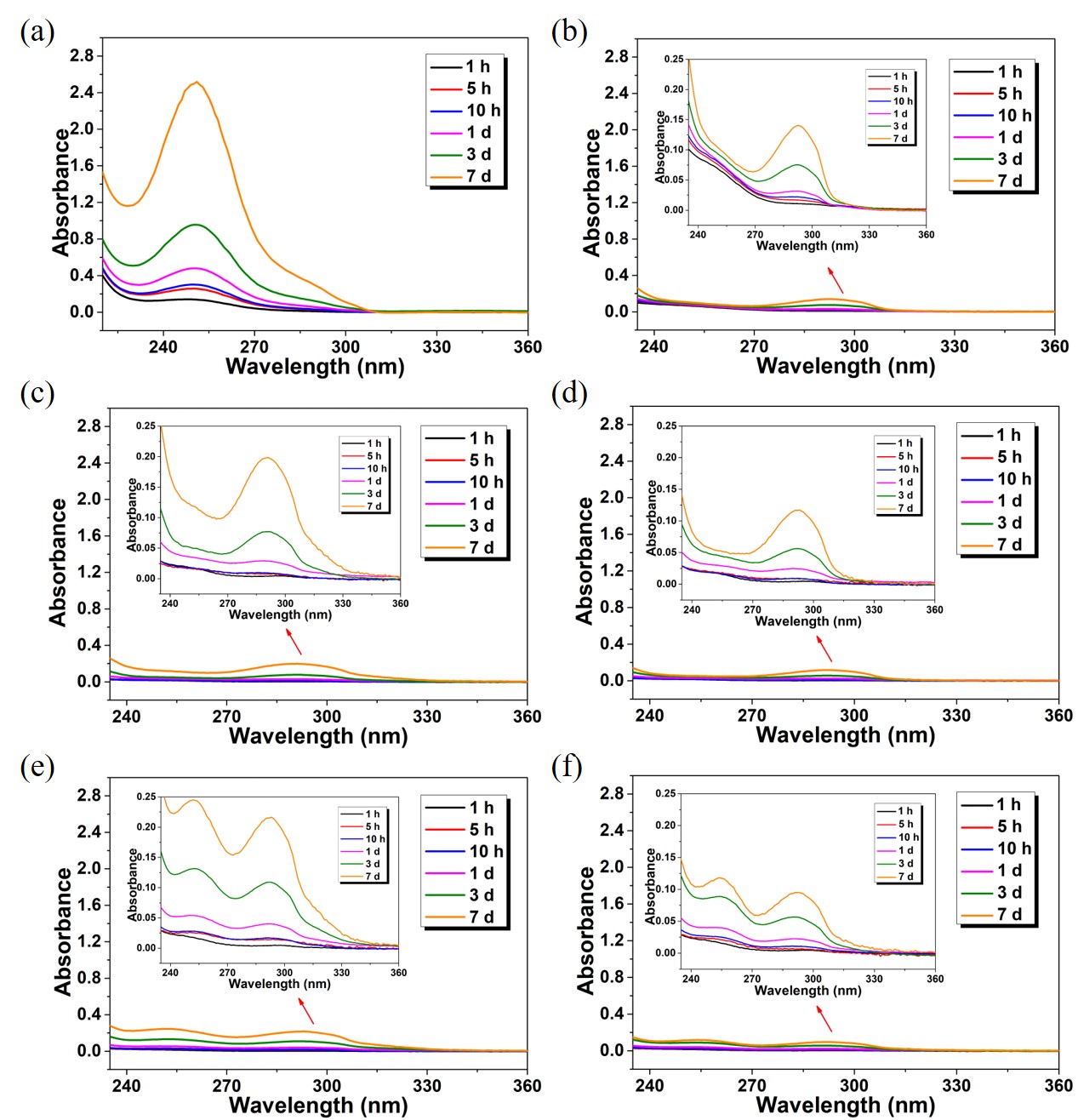


**Figure S23**. Photoinitiator migration rate graph of **BP** (a), **BP-S1** (b), **BP-S2** (c), **BP-S3** (d), **BP-S4** (e), and **BP-S5** (f).

**Table S5**. Migration rate data table of BP and BP-Sn.

| PI | t | A | m_0_ | ε（×10^4^ M^-1^∙cm^-1^） | Migration ratio（%） |
| --- | --- | --- | --- | --- | --- |
| BP | 1 h | 0.13793 | 0.9128 | 2.20 | 2.09 |
|  | 5 h | 0.25618 |  |  | 3.87 |
|  | 10 h | 0.30126 |  |  | 4.56 |
|  | 1 d | 0.47931 |  |  | 7.25 |
|  | 3 d | 0.9563 |  |  | 14.46 |
|  | 7 d | 2.51873 |  |  | 38.09 |
| BP-S1 | 1 h | 0.01009 | 0.9034 | 1.88 | 0.37 |
|  | 5 h | 0.01569 |  |  | 0.58 |
|  | 10 h | 0.02032 |  |  | 0.75 |
|  | 1 d | 0.02911 |  |  | 1.08 |
|  | 3 d | 0.06927 |  |  | 2.57 |
|  | 7 d | 0.12969 |  |  | 4.81 |
| BP-S2 | 1 h | 0.00403 | 0.9158 | 2.35 | 0.13 |
|  | 5 h | 0.00778 |  |  | 0.25 |
|  | 10 h | 0.0096 |  |  | 0.30 |
|  | 1 d | 0.02901 |  |  | 0.92 |
|  | 3 d | 0.07705 |  |  | 2.44 |
|  | 7 d | 0.19854 |  |  | 6.28 |
| BP-S3 | 1 h | 0.00426 | 0.9137 | 2.28 | 0.13 |
|  | 5 h | 0.0086 |  |  | 0.27 |
|  | 10 h | 0.00907 |  |  | 0.28 |
|  | 1 d | 0.02471 |  |  | 0.77 |
|  | 3 d | 0.05558 |  |  | 1.74 |
|  | 7 d | 0.11496 |  |  | 3.60 |
| BP-S4 | 1 h | 0.00432 | 0.9164 | 3.60 | 0.09 |
|  | 5 h | 0.01433 |  |  | 0.28 |
|  | 10 h | 0.01661 |  |  | 0.33 |
|  | 1 d | 0.04009 |  |  | 0.79 |
|  | 3 d | 0.10902 |  |  | 2.15 |
|  | 7 d | 0.2166 |  |  | 4.27 |
| BP-S5 | 1 h | 0.0043 | 0.9203 | 2.92 | 0.11 |
|  | 5 h | 0.00645 |  |  | 0.16 |
|  | 10 h | 0.01077 |  |  | 0.26 |
|  | 1 d | 0.02234 |  |  | 0.55 |
|  | 3 d | 0.0566 |  |  | 1.38 |
|  | 7 d | 0.09491 |  |  | 2.32 |


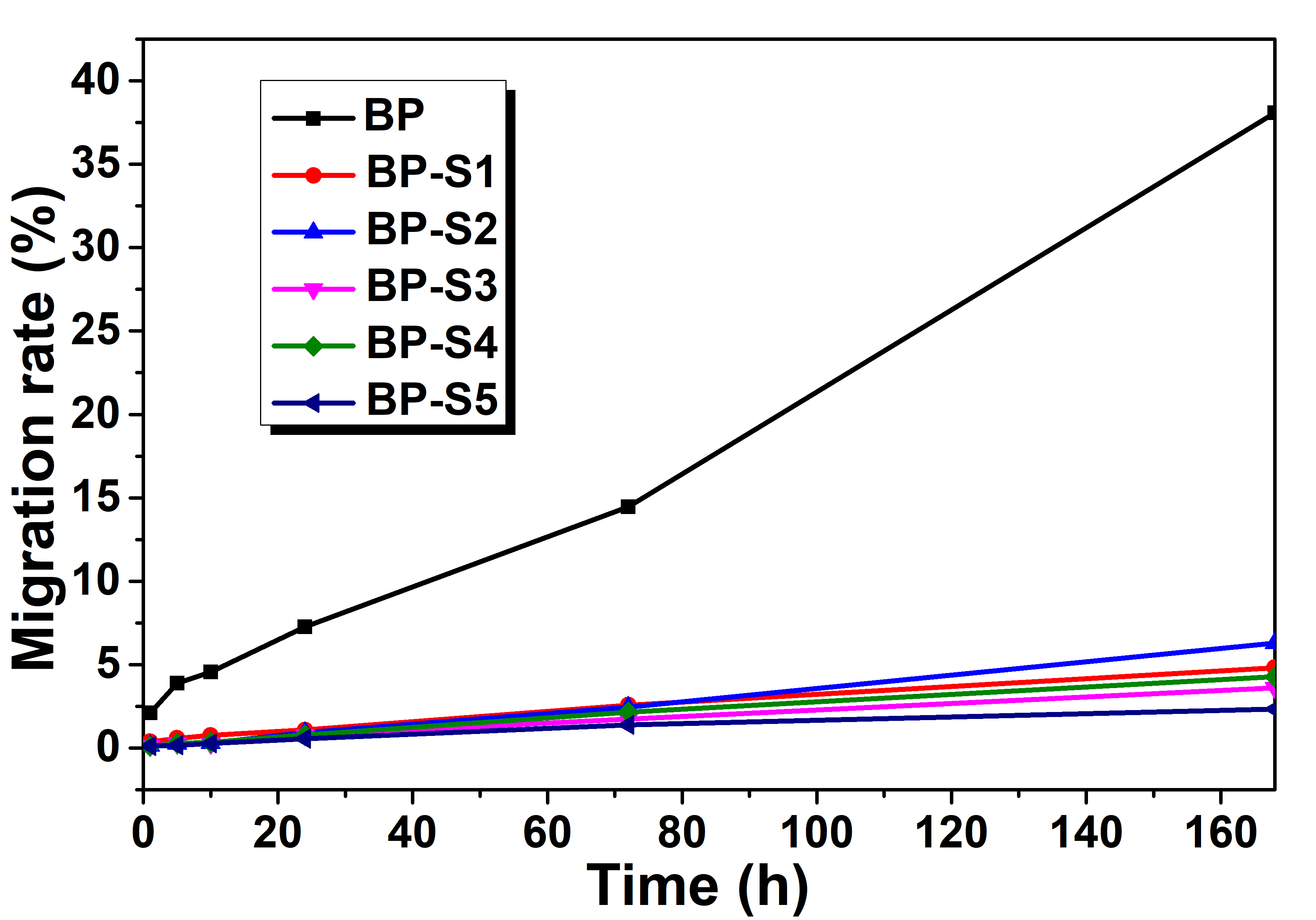
 **Figure S24**. Line chart of photoinitiator migration rate
